# Time-Course Transcriptome Analysis of *Bacillus subtilis* DB104 during Growth

**DOI:** 10.3390/microorganisms11081928

**Published:** 2023-07-28

**Authors:** Ji-Su Jun, Hyang-Eun Jeong, Su-Yeong Moon, Se-Hee Shin, Kwang-Won Hong

**Affiliations:** Department of Food Science and Biotechnology, College of Life Science and Biotechnology, Dongguk University, Goyang-si 10326, Republic of Korea; jiiiisoo@naver.com (J.-S.J.); clove2602@naver.com (H.-E.J.); anstndud2849@naver.com (S.-Y.M.); tpgml426@naver.com (S.-H.S.)

**Keywords:** *Bacillus subtilis* DB104, gene expression, gene regulation, RNA-seq, transcriptome analysis

## Abstract

*Bacillus subtilis* DB104, an extracellular protease-deficient derivative of *B. subtilis* 168, is widely used for recombinant protein expression. An understanding of the changes in gene expression during growth is essential for the commercial use of bacterial strains. Transcriptome and proteome analyses are ideal methods to study the genomic response of microorganisms. In this study, transcriptome analysis was performed to monitor changes in the gene expression level of *B. subtilis* DB104 while growing on a complete medium. Kyoto Encyclopedia of Genes and Genomes (KEGG) analysis, K-mean cluster analysis, gene ontology (GO) enrichment analysis, and the function of sigma factors were used to divide 2122 differentially expressed genes (DEGs) into 10 clusters and identified gene functions according to expression patterns. The results of KEGG pathway analysis indicated that ABC transporter is down-regulated during exponential growth and metabolic changes occur at the transition point where sporulation starts. At this point, several stress response genes were also turned on. The genes involved in the lipid catabolic process were up-regulated briefly at 15 h as an outcome of the programmed cell death that postpones sporulation. The results suggest that changes in the gene expression of *B. subtilis* DB104 were dependent on the initiation of sporulation. However, the expression timing of the spore coat gene was only affected by the relevant sigma factor. This study can help to understand gene expression and regulatory mechanisms in *B. subtilis* species by providing an overall view of transcriptional changes during the growth of *B. subtilis* DB104.

## 1. Introduction

*Bacillus subtilis* is a Gram-positive endospore-forming bacterium and is not considered pathogenic or toxigenic to humans and animals [1,2]. Because of its well-known genetic background and easy genetic manipulation, it is an ideal strain as a host for metabolic engineering or industrial enzyme. *B. subtilis* DB104, a protease-deficient derivative of *B. subtilis* 168, is widely used for recombinant protein expression [3].

Understanding bacterial gene expression is essential for the development of a strain or vector for industrial use. To date, microarray technology has been used to monitor bacterial gene expression. Patten et al. [4] described alternative sigma factor RpoS-dependent gene expression in *Escherichia coli* K-12 using a microarray, while Kitichalermkiat et al. [5] explored the effect of epigallocatechin gallate on *Staphylococcus aureus* gene expression using this technology. During growth in specific conditions, such as with glucose [6] and batch culture [7], transcript profiling was also conducted on *B*. *subtilis* using microarrays. Because the technique relies on hybridization, several disadvantages exist, such as background hybridization that could limit the accuracy and difficulty of designing a large number of probes. RNA-sequencing (RNA-seq) is another technique that provides information about gene expression [8]. RNA-seq based on next-generation sequencing is a widely used transcriptome analysis technology. However, it has limitations of information loss due to the short read length that must be assembled [8,9]. Direct RNA-seq using nanopore is a recently developed technology that can avoid the limitation of the classical RNA-seq [10,11].

RNA-seq and proteomics have become the favored technology platforms for large-scale expression analysis due to the advantages of sensitive and precise detection using technological tools. To date, various omics studies have been conducted on *B. subtilis*. For example, transcriptome or proteome analysis was conducted during specific growth stages, such as early to middle sporulation [12] and germination [13]. Zhang et al. [14] revealed the transcriptional response to cell envelope stress in *B*. *subtilis*. Nicolas et al. [15] explored the regulatory architecture of *B*. *subtilis* using transcriptome analysis in a wide range of conditions. Proteome and transcriptome analyses were applied to define the response to salicylic acid by Van Duy et al. [16] and the response to salt stress by Hahne et al. [17]. Meanwhile, Xu et al. [18] suggested that tmRNA, which liberate ribosomes arrested at mRNA lacking a stop codon, controls biofilm formation using RNA-seq analysis.

However, as it can be seen, most of the omics-based studies on *B. subtilis* have been conducted on a defined medium or at a single time point or specific growth stage. For the immediate application of gene expression data, both in academic research and industrial applications, studies on gene expression and gene function according to their expression patterns are demanded throughout the growth phase of *B*. *subtilis* in a rich medium.

This study uses transcriptome analysis to monitor changes in the gene expression of *B. subtilis* DB104 while growing in Luria–Bertani (LB) medium. First, the growth curve of *B. subtilis* DB104 was prepared, and the total number of bacteria and spores was counted. Then, the growth stage was divided based on optical density and sporulation rate, and changes in RNA expression according to phase were confirmed. In addition, the expression patterns of genes were analyzed, and their functions were investigated. The results of a comprehensive gene expression and functional analysis are expected to increase knowledge about the *B. subtilis* DB104 life cycle.

## 2. Materials and Methods

### 2.1. Bacterial Strain and Growth Conditions

*B. subtilis* DB104 (Δ*npr* Δ*apr*) was routinely grown in LB broth (10 g/L tryptone, 10 g/L NaCl, 5 g/L yeast extract, and pH 7.5). A single colony of *B. subtilis* DB104, grown in LB agar, was inoculated into 5 mL of LB broth and incubated at 30 °C overnight with shaking at 250 rpm. Afterward, the overnight culture was transferred into 50 mL of fresh LB broth in 500 mL baffled flasks at an optical density of 0.1 (OD_600_) and allowed to grow under the same conditions. The cell density was measured by reading the OD_600_ on a spectrometer at various time points. The number of total cells and endospores was also determined. To count the endospores, an aliquot of the culture was centrifugated, resuspended in an equal volume of 1 mg/mL lysozyme, and incubated at 4 °C for 30 min to kill the vegetative cells. 

### 2.2. RNA Isolation, Library Construction, and RNA-Sequencing

RNA was isolated essentially as described [19,20]. The 1.5 mL of *B. subtilis* DB104 culture samples were pelleted by centrifugation at the indicated time points (8, 10, 12, 15, 18, and 24 h). The pellets were treated with 1.5 mg/mL lysozyme in TE buffer [21,22]. Subsequently, 200 μL of TRIzol^®^ Max Bacterial Enhancement Reagent (Life Technologies, Grand Island, NY, USA) was added and the samples were incubated at 95 °C for 4 min. The cell suspensions were mixed with 1 mL of RNAiso Plus (Takara, Otsu, Japan) and total RNA was isolated following the manufacturer’s instructions. Libraries were constructed using a NEBNext Bacteria rRNA Depletion Kit (New England Biolabs, Ipswich, MA, USA) and TruSeq Stranded Total RNA Library Prep Gold Kit (Illumina, San Diego, CA, USA), and 101-bp paired-end reads were generated. Sequencing quality control was conducted using FastQC v0.11.7 (http://www.bioinformatics.babraham.ac.uk/projects/fastqc/, accessed on 15 February 2022). After the library was constructed, clean data were obtained from the raw data by removing the adapter and low-quality reads using Trimmomatic 0.38 (http://www.usadellab.org/cms/?page=trimmomatic, accessed on 15 February 2022). The transcriptome sequences were mapped to the *B. subtilis* subsp. *subtilis* str. 168 genome (GenBank accession number: AL009126.3) using HTseq version 0.10.0 and Bowtie 1.1.2 software. All raw transcriptome data are publicly available in the NCBI Sequence Read Archive (https://ncbi.nlm.nih.gov/sra, accessed on 13 February 2023) database under the accession number PRJNA934277.

### 2.3. Analysis of DEGs

The expression levels of each gene were calculated using the fragments per kilobase of transcript per million fragments mapped (FPKM) value, based on the mapped genes and using Bowtie software. Next, the differentially expressed genes (DEGs) were extracted using edgeR software with a *p*-value < 0.05 and |fold change| ≥ 2. DEGs were obtained from five compared groups (10 h/8 h, 12 h/10 h, 15 h/12 h, 18 h/15 h, and 24 h/18 h). Afterward, all DEGs were grouped into 10 K-mean clusters based on the Z-score. 

### 2.4. Functional Analysis of DEGs

All DEGs were enriched through the Kyoto Encyclopedia of Genes and Genomes (KEGG) database (https://www.genome.jp/kegg, accessed on 20 December 2022). Meanwhile, the DEGs from each cluster that exhibited distinct expression patterns were analyzed with the Biological Networks Gene Ontology (BiNGO) tool to identify their function. The DEGs were annotated using then Gene Ontology (GO) database according to *p*-value < 0.03 and Benjamini–Hochberg-corrected *p*-value < 0.03.

### 2.5. Reverse Transcription-Quantitative Polymerase Chain Reaction (RT-qPCR) Confirmation

The primers used for RT-qPCR are listed in Table S2 (Supplementary File S2). A total of 8 genes (*cotD*, *spoIIID*, *yqfX*, *spmH*, *gsiB*, *frlO*, *cheY*, and *gbsB*) that were found to be most up- and down-regulated DEGs according to fold change were selected and validated by RT-qPCR. One μg of RNA was used to synthesize cDNA using random hexamer (Roche, Base, Switzerland) and M-MLV reverse transcriptase (Promega, Madison, SI, USA), following the manufacturer’s protocols. RT-qPCR was conducted at 95 °C for 10 s, followed by 40 cycles of 95 °C for 5 s and 60 °C for 30 s using Taq Pro Universal SYBR qPCR Master Mix (Vazyme, Nanjing, China) on a CFX Connect Real-Time System (Bio-Rad, Hercules, CA, USA). The RT-qPCR analysis was conducted in triplicate independently. The target genes were normalized against *rpsJ* gene as an internal control. The relative expression levels of the target genes were calculated with the 2^−ΔΔCt^ method [23].

## 3. Results

### 3.1. Cell Growth and Sporulation

**Figure 1:**
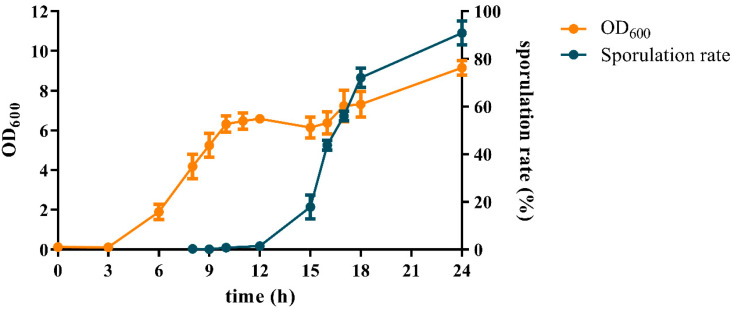
Optical density (OD_600_) and sporulation rate of *B. subtilis* DB104 during growth in LB.

Growth was monitored while cultured in an LB medium to investigate time-related transcriptome changes in *B. subtilis* DB104. First, the growth curve was monitored by optical density measurements at OD_600_ (Figure 1). After 12 h, cell density declined for a while and then from 15 h increased again. Next, sporulation was calculated at various times by plate counting (Figure 1) and observed using an optical microscope (Supplementary File S1: Figure S1). Sporulation was approximately 1% until 12 h, and then rapidly progressed to 18% at 15 h. After 15 h, the sporulation rate constantly increased to 90% at 24 h. Therefore, *B. subtilis* DB104 growth could be divided into two parts: exponential growth and sporulation with a transition point at 12 h.

### 3.2. RNA Sequencing and Analysis of Differentially Expressed Genes (DEGs)

Cell samples from six-time points were obtained based on the growth curve: the middle of exponential growth (8 h), the end of exponential growth (10 h), the beginning of sporulation (12 h), the former part of sporulation (15 h), the latter part of sporulation (18 h), and the end of sporulation (24 h). At each time point, the total RNAs were extracted from three independent cultures and used for RNA-seq. The transcriptome sequences were mapped against the *B. subtilis* subsp. *subtilis* str. 168 reference genome (GenBank accession number: AL009126.3). A summary of the resulting sequence data is shown in Table S1 (Supplementary File S2). The filtered clean reads ranged from 21,238,576 to 35,756,134. The Q20 values were greater than 98.19%, Q30 values were greater than 94.16%, and the GC contents were greater than 44.58% in each sample.

**Figure 2:**
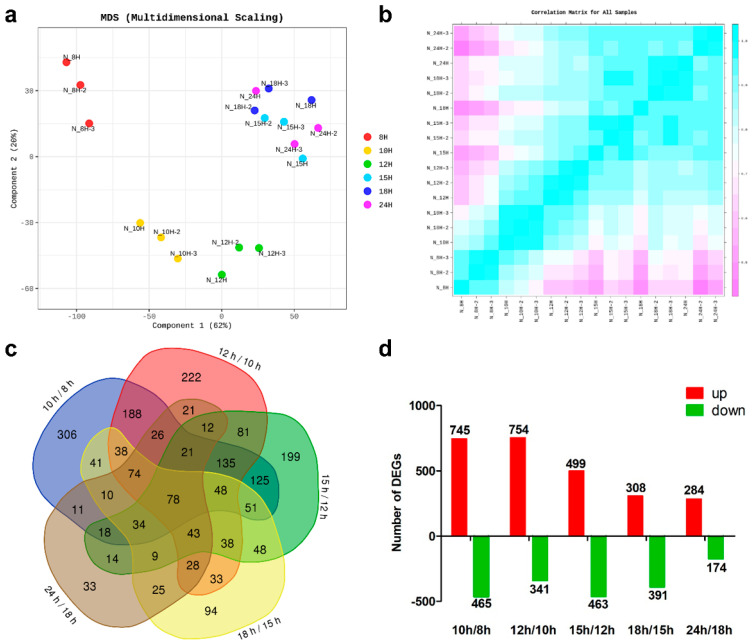
Analysis of the transcriptomic data. (**a**) Multidimensional scaling plot and (**b**) Pearson correlation coefficient of all samples. (**c**) Venn diagram analysis and (**d**) the numbers of DEGs identified by pairwise times with a |fold change| ≥ 2.

To ensure the reliability of the transcriptome analysis, multidimensional scaling and the Pearson correlation coefficient were used to confirm the differences among all samples (Figure 2a,b). According to the results, the 10 h and 12 h groups had similar RNA expression patterns. The 15 h, 18 h, and 24 h groups were similar, whereas the 8 h group was relatively separated from the other groups. This suggests that the transcription profile of *B. subtilis* DB104 was altered, depending on the growth stage.

With a *p*-value < 0.05 and |fold change| ≥ 2 as the criteria, a total of 2122 DEGs were identified in all the comparison groups. The numbers of DEGs identified between the various time points during the growth of *B. subtilis* DB104 were analyzed using a Venn diagram (Figure 2c). The number of genes that were up- or down-regulated in each compared group is shown in Figure 2d. The results show that more transcriptome changes occurred during exponential growth to the former part of sporulation than the latter part and end of sporulation. In general, the number of up-regulated DEGs was higher than that of down-regulated DEGs.

### 3.3. DEGs with the Highest Expression Levels and Fold Changes

To identify the most highly expressed genes, expression levels at all time points were calculated based on transcripts per millions mapped reads (TPM). The 10 most highly expressed DEGs are listed in Table 1. Genes encoding various spore coat proteins, such as *cotX*, *cotY*, and *cotZ*, showed high TPM values. Other extreme TPM values were observed for genes related to the spore of *B. subtilis*, such as *gerE*, s*afA*, *cgeA*, and *cgeB*. Most of these genes were strongly expressed after 12 h. Among the genes listed in Table 1, only *sdpC* and *skfA* were highly expressed during exponential growth, and their expressions were down-regulated after 12 h. The DEGs with the highest fold-change values were also confirmed, and the top 10 of them are listed in Table 2. The most up-regulated genes were mostly related to sporulation and germination, such as the spore coat proteins *cotD* and *cotG* and the germination proteins *yqfX* and *yqzD*. In contrast, the most down-regulated genes were associated with a higher variety, such as response to stress (*gsiB* and *gabT*), chemotaxis (*cheY*), and metabolism (*gabT*, *gbsB*, *gbsA*, and *xpt*). Furthermore, the down-regulated genes showed a much smaller fold-change value than the up-regulated genes. The highest expression changes did not appear after 18 h. Altogether, these results show that sporulation-related genes are highly expressed and regulated, and sharp changes in gene expression mainly occur up to the former part of sporulation (15 h) during *B. subtilis* DB104 growth. 

### 3.4. KEGG Pathway Enrichment Analysis

KEGG pathway analysis was performed to elucidate changes in pathways. DEGs in each compared group were divided into two groups consisting of up- or down-regulated cases and were analyzed separately. The total number of DEGs involved in each pathway was calculated. Table 3 shows the top 10 KEGG pathways with the number of DEGs involved. The DEGs were related to metabolic pathways (bsu01100), microbial metabolism in diverse environments (bsu01120), biosynthesis of amino acids (bsu01230), and butanoate metabolism (bsu00650) in both cases, which suggests that metabolic pathway changes are significant throughout growth. In addition, it was noticed that the genes involved in ABC transporters (bsu02010) were down-regulated up to the beginning of sporulation (12 h) and up-regulated from the latter part of sporulation (18 h). 

### 3.5. Cluster Analysis

**Figure 3:**
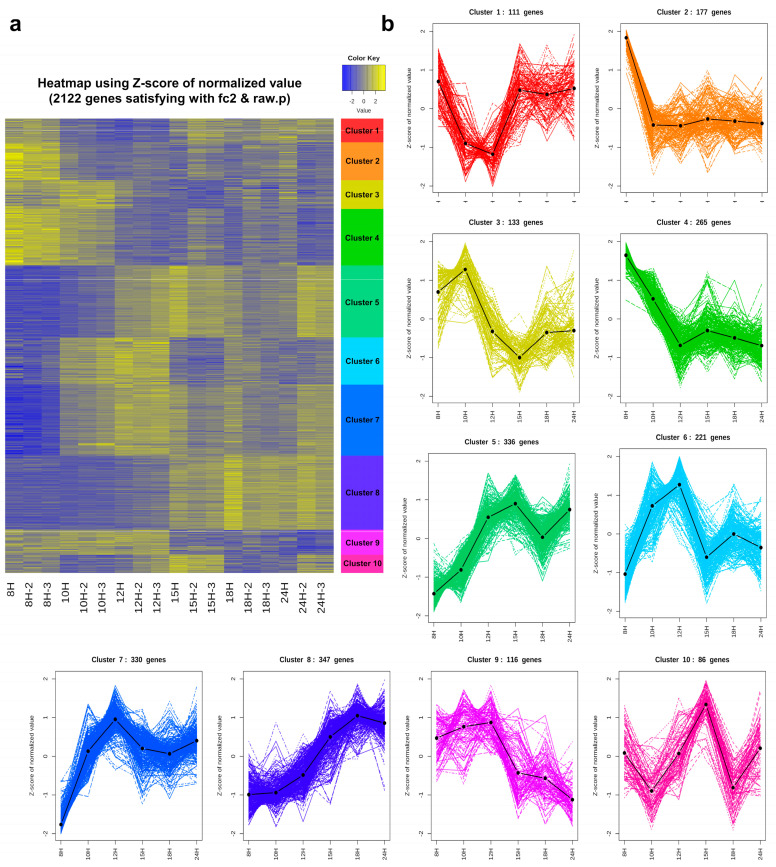
K-mean clustering analysis of differentially expressed genes (DEGs). (**a**) Heatmap of each cluster over time (columns) for all DEGs. (**b**) The expression patterns of each cluster.

Based on the Z-score, a K-mean cluster analysis was performed to further analyze the DEG expression patterns further. Ten distinct clusters were obtained, and their expression levels across growth were presented by a heatmap (Figure 3). Each cluster exhibits various expression patterns over time; in cluster 1, gene expression was decreased from 8 to 12 h, while it was increased thereafter until being equal to 8 h (*n* = 111 genes); in cluster 2, expression sharply decreased at 10 h and remained at that level (*n* = 177 genes); in cluster 3, gene expression increased slightly at 10 h and decreased with a nadir at 15 h, while it was slightly recovered thereafter (*n* = 133 genes); in cluster 4, the expression of the included genes was down-regulated to 12 h and then remained at a similar level (*n* = 265 genes); in cluster 5, the expression increased in a time-dependent manner with the zenith at 15 h (*n* = 336 genes); in cluster 6, 7, and 9, gene expression was up-regulated from 8 to 12 h, and then decreased at 15 h (*n* = 221, 330, and 116, respectively); in cluster 8, expression levels increased in a time-dependent manner with maximum expression at 18 h (*n* = 116 genes); and in cluster 10, genes were expressed in a zig-zag manner with the deepest parts at 12 and 18 h and the highest point at 15 h (*n* = 86 genes). The genes belonging to each cluster are listed in Supplementary File S3. 

### 3.6. Functional Enrichment Analysis of Each Cluster

Gene ontology (GO) enrichment analysis was conducted to confirm the biological function of genes belonging to each cluster that showed different expression patterns over time. The genes in each cluster were classified into biological process (BP), molecular function (MF), and cellular component (CC). The GO terms that were significantly enriched and their relationships are shown in Tables S3–S11 (Supplementary File S2) and Figures S2–S10 (Supplementary File S1).

#### 3.6.1. Genes That Are Down-Regulated at the End of Exponential Growth: Cluster 2

The 177 genes in cluster 2 showed the high expression levels in the middle of exponential growth (8 h) but decreased expression from 10 h at the end of exponential growth (Figure 3). DEGs belonging to this cluster were classified into 130 GO terms. The enriched GO terms are listed in Table S4 (Supplementary File S2), and their relationship is shown in Figure S2 (Supplementary File S1). Among them, the terms related to BP were the most common with 96, and mostly consisted of various metabolic processes (GO:0008152), such as ribonucleotide metabolic process (GO:0009259), amine catabolic process (GO:0009310), and organic acid metabolic process (GO:0006082). Terms, such as signaling (GO:0023052), secretion (GO:0046903), locomotion (GO:0040011), and localization (GO:0051179), were also found to be significantly enriched terms. Sixteen GO terms were related to CC and associated with the flagellum (GO:0019861).

#### 3.6.2. Genes That Are Down-Regulated from the Beginning of Sporulation: Clusters 3 and 4

Genes belonging to clusters 3 and 4 were expressed at a low level from 12 h when sporulation begins to 24 h when sporulation ends (Figure 3). Among them, 133 DEGs belonging to cluster 3 were classified into 90 GO terms. The enriched classified GO terms are listed in Table S5 (Supplementary File S2), and their relationship is shown in Figure S4 (Supplementary File S1). The 79 BP terms include subterms of metabolic processes, such as cellular amino acid biosynthetic process (GO:0008652) and cellular metabolic process (GO:0044237), and subterms of developmental process, such as sporulation (GO:0043934) and cell differentiation (GO:0030154). In addition, GO terms, such as urea metabolic process (GO:0019627), secretion (GO:0046903), response to stimulus (GO:0050896), and cell communication (GO:0007154), were also revealed as significantly enriched terms. The 256 DEGs of cluster 4 were significantly enriched in 69 BP, 25 MF, and 15 CC terms. The enriched terms are listed in Table S6 (Supplementary File S2), and their relationship is shown in Figure S5 (Supplementary File S1). The DEGs enriched in BP were related to tricarboxylic acid cycle (GO:0006099), chemical homeostasis (GO:0048878), chemotaxis (GO:0006935), response to stimulus (GO:0050896), and localization (GO:0051179). In addition to protein complex (GO:0043234) and extracellular region (GO:0005576), the DEGs enriched in CC were especially related to flagellum (GO:0019861).

#### 3.6.3. Genes That Are Turned on at the Beginning of Sporulation and then Turned off in the Middle: Clusters 5, 6, 7, and 9

Four out of ten clusters showed a pattern where expression decreased from 15 h or 18 h (middle of sporulation). Among them, clusters 6 and 7 showed similar expression patterns, with expression at a low level in the exponential phase and then increased until sporulation began; however, differences in expression level were observed after 15 h. The 221 DEGs belonging to cluster 6 were classified into only 21 BP terms. The 330 DEGs belonging to cluster 7 were classified into 30 BP, 8 MF, and 2 CC terms. The enriched terms in each cluster are listed in Tables S8 and S9 (Supplementary File S2), and their relationships are shown in Figures S7 and S8 (Supplementary File S1), respectively. The BP-enriched DEGs of clusters 6 and 7 were mainly related to developmental process (GO:0032502), such as sporulation (GO:0043934) and cell differentiation (GO:0030154), and some other metabolic processes. The expression pattern of cluster 9 differed in that it had a high expression level from exponential growth, although it decreased from 15 h. As a result of annotating 116 DEGs belonging to cluster 9 into the GO database, there was no significant term that satisfied the *p*-value < 0.03, and the Benjamini–Hochberg-corrected *p*-value < 0.03. Unlike other clusters, DEGs of cluster 5 increased expression up to 15 h and then decreased from 18 h. The 336 DEGs belonging to cluster 5 were classified into nine BP terms and one CC term. The enriched terms are listed in Table S7 (Supplementary File S2), and their relationship is shown in Figure S6 (Supplementary File S1). BP terms were focused on developmental processes (GO:0032502), such as sporulation (GO:0043934) and cell differentiation (GO:0030154).

#### 3.6.4. Genes That Are Up-Regulated in the Middle of Sporulation: Clusters 1 and 8

Genes belonging to cluster 1 were down-regulated at 12 h, but their expression increased from 15 h, the former part of the sporulation (Figure 3). The 111 DEGs of cluster 1 were classified into 53 GO terms. The enriched terms are listed in Table S3 (Supplementary File S2), and their relationship is shown in Figure S2 (Supplementary File S1). In cluster 1, most DEGs were enriched in BP terms, including metabolic processes such as tryptophan metabolic process (GO:0006568) and lipoteichoic acid biosynthetic process (GO:0070395) in addition to cell wall organization or biogenesis (GO:0071554), localization (GO:0051179), and small molecule metabolic process (GO:0044281). Of the seven MF terms, terms related to transporter activity (GO:0005215) were the most common, appearing in four. Terms enriched in this cluster were classified with relatively high *p*-values. In contrast, terms classified as enriched in cluster 8 had low *p*-values and were concentrated in developmental processes (GO:0032502). DEGs belonging to cluster 8 steadily increased expression from the beginning to the latter part of sporulation. The 347 genes belonging to this cluster were enriched in 15 BP terms. The enriched terms are listed in Table S10 (Supplementary File S2), and their relationship is expressed in Figure S9 (Supplementary File S1). In addition to the subterm of the developmental process (GO:0032502), these terms were also found to be significant in terms related to the organic cation transport (GO:0015695).

#### 3.6.5. Genes That Are Transiently Turned on at the Former Part of Sporulation: Cluster 10

The genes belonging to cluster 10 were specifically turned on at 15 h (the former part of sporulation) and then down-regulated. The enriched terms are listed in Table S11 (Supplementary File S2), and their relationship is shown in Figure S10 (Supplementary File S1). The 86 DEGs in this cluster were enriched mainly for BP terms related to metabolic process, such as lipid catabolic process (GO:0016042) and organic acid catabolic process (GO:0016054), or monosaccharide transport (GO:0015749).

### 3.7. Sigma Factors

Various alternative sigma factors exist in *B. subtilis* and are transcribed by recognizing the promoter of the specialized gene set. By analyzing the expression patterns of genes regulated by each alternative sigma factor, their expression patterns were confirmed with clusters revealed through K-mean cluster analysis. The genes regulated by each sigma factor were classified using the DBTBS database [24]. σ^A^ is a housekeeping sigma factor that regulates gene expression during the exponential phase and early sporulation in *B. subtilis*. The gene that encodes σ^A^, *sigA*, was expressed highly during whole growth, especially at 12 h, where sporulation started (Table 4). Genes controlled by σ^A^ were found to be distributed across all clusters. In *B. subtilis*, σ^B^ is a sigma factor that regulates various general stress genes. Clusters 6, 7, and 9, which have the highest expression at the transition point of 12 h, commonly contain many genes controlled by this sigma factor. However, the gene that encodes σ^B^, *sigB*, was expressed constantly during growth (Table 4). σ^D^ is a sigma factor that regulates genes related to flagellar gene expression, chemotaxis, autolysis, and motility in *B. subtilis*. Cluster 2, whose expression was suppressed at the end of exponential growth, contained many genes controlled by σ^D^, including *sigD*. *sigD* (the gene for σ^D^) is a component of the *fla*-*che* operon, which consists of more than 30 genes and can self-activate. Many genes comprising this operon were included in cluster 2, including the genes encoding the rod protein *flgC* and the hook proteins *flgE*, *cheA*, *cheB*, and *cheW*, which comprise the flagellar of *B. subtilis*. In addition, *flhO* and *flhP*, which do not belong to this operon but encode the proteins that make up the flagellar rod, were also included in this cluster. Cluster 4, which exhibits a pattern where expression decreases as sporulation starts, also contained many genes controlled by σ^D^, similar to cluster 2. Among the *fla*-*che* operon genes, genes in cluster 4 included the genes encoding the hook proteins *flgL* and *flgK* and the rod protein *flgB*. In addition, the genes encoding flagellar assembly protein genes *fliS* and *fliT*, the flagellin protein *hag*, and the flagellar stators *motA* and *motB* are not part of the *fla*-*che* operon and are controlled by σ^D^, belonging to this cluster. Among the genes controlled by σ^D^, genes related to autolysis, such as *lytB*, *lytC*, and *lytF*, were included in cluster 4. Genes regulated by the sigma factor associated with sporulation were included in the cluster with the highest expression at 15 h or 18 h. The genes controlled by σ^E^ and σ^F^, which are early sporulation-related sigma factors, were included in cluster 7. The DEGs controlled by σ^E^ that regulate early sporulation genes in mother cells were also included in cluster 6. In contrast, genes controlled by σ^K^, a sigma factor related to late sporulation gene expression in the forespore, and σ^G^, sigma factor related to late sporulation gene expression in the mother cell, were predominantly included in clusters 8 and 5, respectively. The genes that encode early sporulation-specific sigma factors, *sigE* and *sigF,* were up-regulated at 10 h (Table 4). However, the genes that encode late sporulation-specific sigma factor, *sigG,* were up-regulated from 10 h. The other late sporulation-specific sigma factor σ^K^, which consists of two truncated genes, *sigKc* and *sigKn* [25], are highly expressed from 12 h. General stress protein genes, such as *yceD*, *yceE*, *yceF*, and *yceG*, are controlled by σ^W^, an extra-cytoplasmic function sigma factor and were included in clusters 4 or 9. The gene for σ^W^, sigW, was also included in cluster 9, and its expression was down-regulated from 15 h (Table 4). Among the regulons of σ^L^, known to be involved in degradative enzyme expression, only the acetoin dehydrogenase operon genes (*acoABCL*) were classified as cluster 8, and the rest did not belong to any cluster. The gene that encodes σ^L^, *sigL*, was up-regulated until 10 h and then down-regulated.

Several other factors, such as σ^I^, σ^M^, σ^X^, σ^Y^, and YlaA, along with the sigma factors described above, regulate gene expression in *B. subtilis*. The *dhb* operon genes (*dhbACEBF* and *mbtH*) are controlled by σ^I^ and were included in cluster 9. Some flavodoxin genes (*fldN*, *ykuO*, and *fldP*), which are controlled by the same sigma factor, were included in cluster 10. σ^X^ regulon has *dlt* operon genes, such as *dltABCDE*, belonging to cluster 1 and the *efe* operon genes (*efeUBO*) and *yce* operon genes (*yceDEFGH*) belonging to cluster 4. In addition, some of the genes controlled by σ^X^ belong to cluster 7 or cluster 6. YlaC, known to regulate genes associated with oxidative stress, regulates the *yla* operon to which it belongs, and genes belonging to this operon (*ylaACD*) were included in cluster 7 (the *ylaB* gene in *yla* operon was not mapped in this study).

### 3.8. Flagella

**Figure 4:**
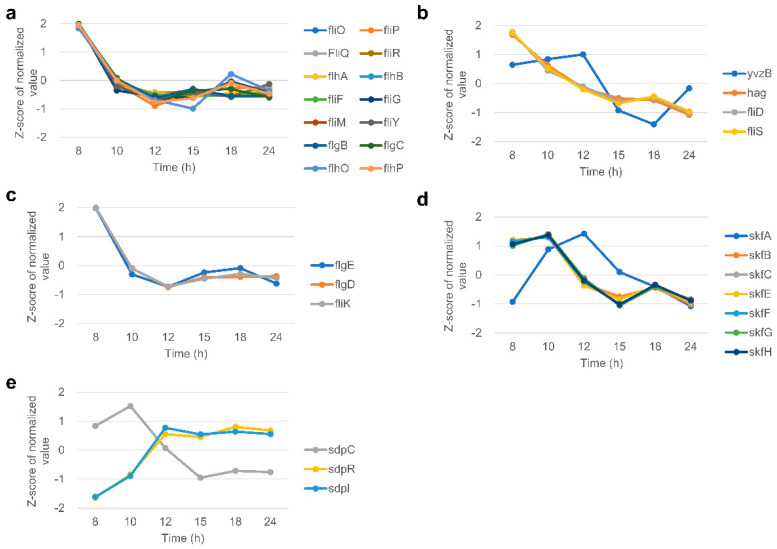
Expression patterns of DEGs. Z-score value of (**a**) basal body, (**b**) filament, and (**c**) hook genes of *B*. *subtilis* flagella. (**d**) Z-score value of the *skf* operon. (**e**) Z-score value of the *sdpABC* and *sdpRI* operons.

Among the movement of bacteria, swimming and swarming are the most representative. *B. subtilis*, a flagellated bacterium, can swim in liquids and swarm on solid surfaces through flagella. Therefore, the analysis of the flagellar gene could help to understand the mobility of this strain. The expression values of some flagellar structure genes, such as *fliO*, *fliP*, *flgB*, *fliD*, and *flgD*, are shown in Figure 4a–c and Table S12 (Supplementary File S2), which were classified as cluster 2 or 4 through K-mean clustering analysis. These genes remained down-regulated from the end of exponential growth (10 h) or the transition point (12 h). On the other hand, *yvzB* showed a different expression pattern from other flagellar genes, maintaining its expression level until 12 h, when sporulation began, and then down-regulated from the former part of sporulation (15 h). 

### 3.9. Sporulation and Spore Coat

**Figure 5:**
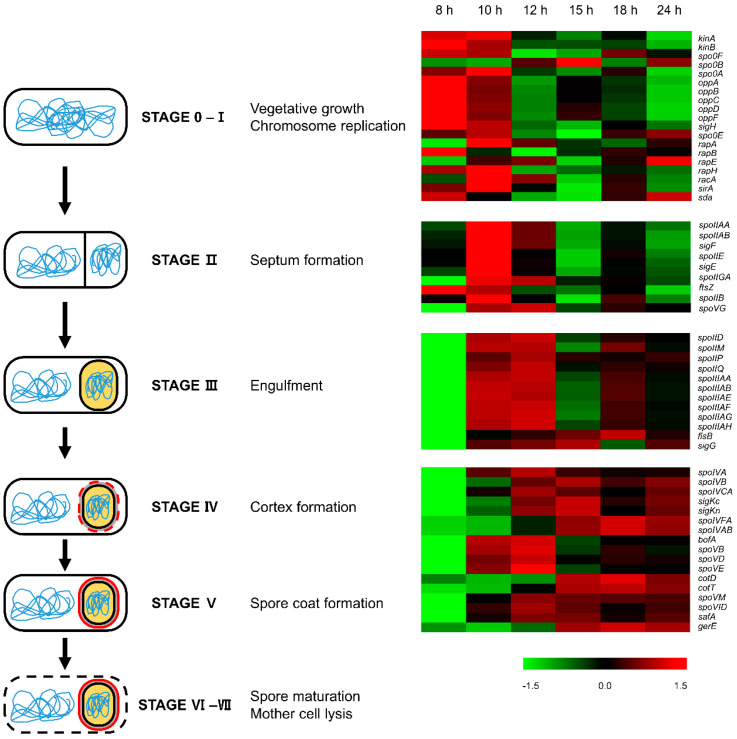
Sporulation stage of *B. subtilis* and expression patterns of genes involved in sporulation. Gene expression is visualized as the Z-score. The red and green colors represent the up-regulated and down-regulated genes, respectively.

The expression patterns of the gene involved in each sporulation stage are shown in Figure 5. In stage 0, cannibalism delays entering the sporulation process (in the next section). Most genes related to sporulation stage 0 and I were up-regulated at the exponential growth (8 h and 10 h). When septum is formed, many genes expressed in stage II were mostly up-regulated at 10 h (the end of exponential growth). Most of the genes expressed when stage III is completed also showed similar expression patterns to those of stage II but remained at a highly expressed level until 12 h (the beginning of sporulation). Most genes expressed during stages VI and V, in which the cortex and spore coat are formed and assembled, were up-regulated after 12 h, and they remained consistently high until 24 h when sporulation was completed.

**Figure 6:**
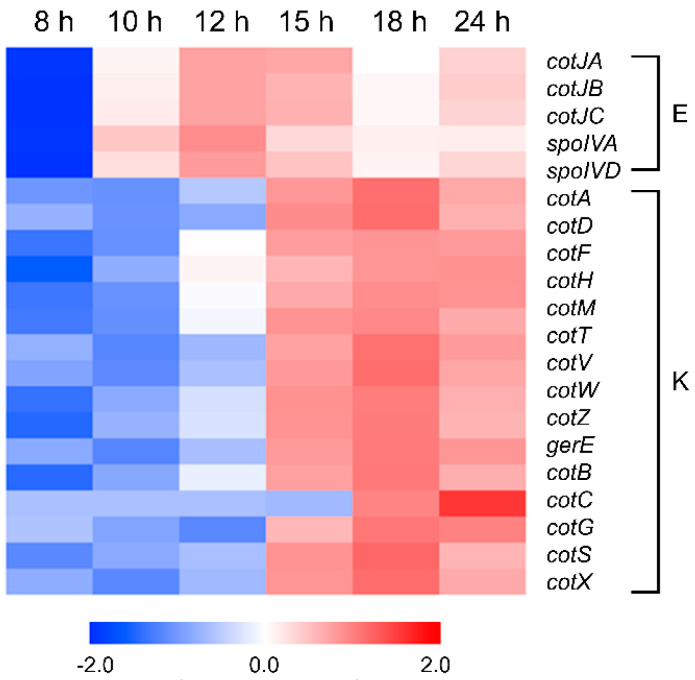
Expression patterns of the genes involved in spore coat expression. Gene expression is visualized as the Z-score. The red and blue colors represent the up-regulated and down-regulated gene, respectively. E represents the σ^E^-dependent genes. K represents the σ^K^-dependent genes.

After spore engulfment was completed, σ^E^ first induced the expression of the spore coat proteins in the mother cell. The expression value of representative spore coat proteins of *B. subtilis* is shown in Figure 6 and Table S12 (Supplementary File S2). The σ^E^-induced spore coat protein genes (*cotE*, *cotJA*, *cotJB*, and *cotJC*) and spore morphogenic genes (*spoIVA* and *spoVID*) became up-regulated from 10 h at the end of exponential growth (Figure 6; the *cotE* gene was not mapped in this study). Afterwards, most of the spore coat protein genes, such as *cotA*, *cotD*, *cotF*, *cotH*, *cotM*, *cotT*, *cotV*, *cotW*, *cotY*, *cotZ*, *cotB*, *cotG*, *cotS*, and *cotX*, and the spore coat gene transcription regulator gene *gerE* were up-regulated by σ^K^ from 15 h, the former part of sporulation (Figure 6). 

### 3.10. Cannibalism

At the beginning of sporulation in *B. subtilis* (stage 0), cannibalistic behavior occurs to delay entering this energy-consuming process. The *skf* (sporulation killing factor) and *sdp* (sporulation delay protein) operons become involved in cannibalism. Among the *skf* operon genes, *skfA*, which encodes the sporulation killing factor itself, was strongly expressed in 10 h at the end of exponential growth and 12 h at the beginning of sporulation then decreased (Figure 4d). However, except for *skfA*, the remaining *skf* operon genes showed a different pattern in which expression decreased from 12 h. In addition, the expression of those genes was significantly lower than that of *skfA* (Supplementary File S2: Table S13). Among the *sdpABC* operon, only the *sdpC* gene belonged to DEGs. Its expression was highest at 10 h and decreased sharply from 12 h when sporulation began (Figure 4e). Conversely, the expression of the *sdpRI* immunity operon continued to be up-regulated from 12 h to 24 h. The *sdpA* and *sdpB* genes of the *sdpABC* operon and the *sdpI* and *sdpR* genes of the *sdpRI* operon showed very-low expression values compared to *sdpC* (Supplementary File S2: Table S13).

### 3.11. RNA-Seq Data Validation by RT-qPCR

**Figure 7:**
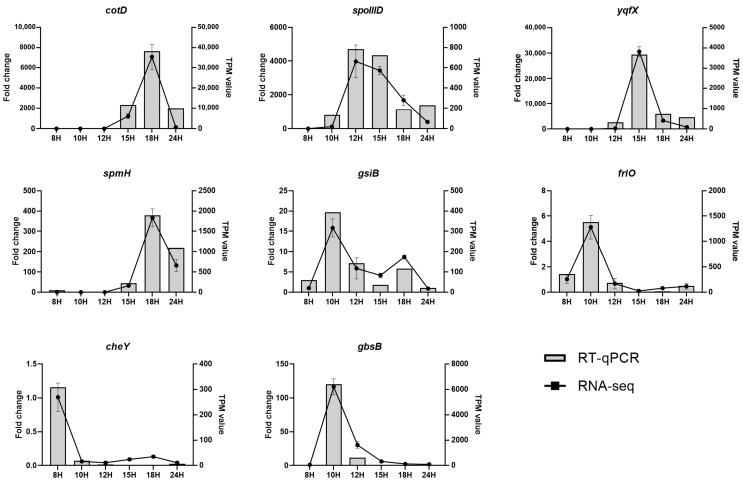
Real-time quantitative PCR validation of RNA-seq data for most up- and down-regulated DEGs. The right and left y-axis represent the TPM value of RT-qPCR and fold change of RNA-seq, respectively. The fold-change values were generated for RT-qPCR samples by comparing the expression of genes at each timepoint vs. at 8 h. RT-qPCR analysis for RNA expression of target genes was normalized against *rpsJ*. RT-qPCR data are presented as the mean ± standard deviation (*n* = 3).

To validate the accuracy of transcriptome data, RT-qPCR analysis was conducted. Table 2 shows the top 10 up- and down-regulated DEGs according to fold change. Therefore, eight genes included in Table 2 (*cotD*, *spoIIID*, *yqfX*, *spmH*, *gsiB*, *frlO*, and *cheY*) were selected for validation by RT-qPCR. RNA samples from six-time points were used as templates. The results are shown in Figure 7, indicating that the data from RNA-seq and RT-qPCR analysis were consistent.

## 4. Discussion

This study investigated gene expression changes in *B. subtilis* DB104 during growth in a LB complete medium through RNA-seq. The simultaneous application of several analysis methods (DEG analysis, KEGG analysis, K-mean cluster analysis, GO analysis, and sigma factor function analysis) allowed the expression patterns of genes and their function to be revealed. We described gene expression changes using RNA-seq analysis, providing genome-wider information compared to using microarray analysis [6,7]. 

When bacterial growth becomes limited due to nutrient depletion, exponential growth ceases and the bacteria enter the stationary phase. The period between these two phases is called a transition point [26,27]. This transition point has been also observed in *B. subtilis*, and various metabolic pathway changes occur here [6,7]. In the present study, 12 h was found to be the transition point at which exponential growth ceases and the stationary phase starts (Figure 1). In addition, most of the highest fold changes in DEGs were found at this time point (Table 2). In addition, KEEG analysis revealed that alterations occurred in metabolic pathways around the transition point. The metabolic pathway (bsu01100) was up-regulated until 12 h, and some of the DEGs involved in this pathway started to become down-regulated from 15 h (Table 3). Previous studies showed that the metabolic rate decreased when the stationary phase started in the growth of *B. subtilis* [28,29]. The other enriched metabolic pathways, such as microbial metabolism in diverse environments (bsu01110) and the biosynthesis of secondary metabolites (bsu01110), were up-regulated to 12 h.

ABC transporters are responsible for transporting a wide range of substrates, such as amino acids, sugars, and organic acids, across the cell membrane through ATP hydrolysis and can perform various functions. Among the pathways enriched by KEGG analysis, only the DEGs involved in ABC transporters (bsu02010) were down-regulated before the transition point and subsequently up-regulated. A possible reason for this may be that a specific ABC transporter is required to initiate sporulation. The ABC transporter gene *ftsEX*, whose transcription levels were down-regulated at 10 h in this study (Supplementary File S2: Table S13), plays a role in spore formation initiation [30]. In addition, the other ABC transporter genes *oppA*, *oppB*, *oppC*, *oppD*, and *oppF*, which are also involved in the initiation of sporulation [31,32], were down-regulated at 12 h (Supplementary File S2: Table S13). The fact that this system is related to stress responses, such as osmotic stress, could be another reason. When bacteria reach the stationary phase, they undergo various metabolism changes and become resistant to several stresses [7,33]. Corresponding to this, the genes for the osmoprotectant ABC transporters *opuBA*, *opuBB*, and *opuBC* were up-regulated at 18 h but were down-regulated at 12 h.

Diverse transcription regulators exist in *B. subtilis*. Among them, various alternative sigma factors, which allow RNA polymerase to recognize the promoter sequence rapidly and specifically, are essential for sporulation, stress response, chemotaxis, and motility [34]. Therefore, the sigma factors are a significant tool for understanding the gene expression of *B. subtilis*. To identify the different expression patterns of DEGs and their functions, K-mean clustering was coupled with GO and sigma factor analysis. In this study, the DEGs were divided into 10 clusters whose expression profiles could be redivided into five patterns in accordance with growth stage. Because gene expression was examined according to six-time points, the 10 clusters were divided again, as described above (Section 3.6). 

The first group of clusters, which contains only cluster 2, has a pattern specifically expressed only in the middle of exponential growth. The DEGs belonging to cluster 2 were enriched in most terms. The relationship between them was also complicated, as shown in Figure S3 (Supplementary File S1), indicating that genes for maintaining various biological functions are highly expressed in exponential growth and subsequently turned off. Genes transcribed by σ^A^- or σ^D^-dependent RNA polymerase were the most prevalent among the 177 DEGs in cluster 2. *B. subtilis* σ^A^, similar to its *E*. *coli* counterpart σ^70^, is the primary sigma factor involved in the transcription of most housekeeping genes [35]. This sigma factor also participates in the transcription of the early sporulation genes [36,37] and stress-inducible genes [38,39]. It has been found that the expression of *sigA* (gene for σ^A^) remains constantly high during the whole growth of *B*. *subtilis* DB104 (Table 4). In addition, the primary sigma factor, σ^A^ in *B*. *subtilis*, usually transcribes genes with other sigma factors [40,41]. This could be why σ^A^-dependent genes were evenly distributed across all clusters in the transcriptome data. Many genes included in clusters 1 to 10 were transcribed by σ^A^-dependent promoter. However, this result does not correspond with that of a previous study, which found that the expression of σ^A^-dependent genes is turned off in the stationary phase [6]. In contrast, Blom et al. [7] showed results consistent with this research and observed that the SigA regulon is overrepresented in the exponential growth phase but could be expressed after the transition point. Furthermore, results from this study suggest that σ^A^-dependent genes have various expression patterns because σ^A^ works both alone and jointly with other sigma factors. *B. subtilis* σ^D^ controls genes related to flagellar [42], autolysis [43], chemotaxis, and motility [44]. σ^D^-dependent genes were included predominantly in cluster 2 in addition to cluster 4 in the second cluster group (genes that are down-regulated from the beginning of sporulation). With corresponding data, the GO term “flagellum (GO:0019861)” was enriched in clusters 2 and 4. As previously stated, *yvzB*, the flagella structure gene, began to decline after the transition point, but remained highly expressed until sporulation began (Figure 4a–c). This may be because this gene is a putative filament gene, and it is unknown whether it contains the proteins that make up the filament structure. Mukherjee and Kearns [45] suggested that flagella synthesized in a mother cell could function in a granddaughter cell. The results from this study showed that flagellar structural genes are down-regulated during the stationary phase in *B. subtilis* DB104, but further research is needed to determine whether this weakens the swarming/swimming mobility. Meanwhile, among the genes controlled by σ^D^, autolysis-related genes were included in cluster 4 rather than cluster 2, indicating that autolysin expression continued later than that of flagella proteins. The *sigD* (gene for σ^D^) was also included in cluster 2, and the expression level was down-regulated from 10 h (Table 4). This result indicates that σ^D^ produced in the middle of exponential growth remains and functions afterward. The DEGs belonging to clusters 3 and 4 showed a high expression level in exponential growth, similar to genes belonging to cluster 2. However, the difference is that they maintained a high expression afterward and then showed a low expression from the beginning of sporulation. They were also classified as diverse and complex terms (Tables S5–S7 in Supplementary File S2, Figures S4 and S5 in Supplementary File S1). Therefore, these results show that genes for maintaining various biological functions are highly expressed during exponential growth, and some are up-regulated even after exponential growth is over.

An extracellular RNA polymerase sigma factor of *B. subtilis*, σ^W^, is involved in the response to cell envelope stress, such as to antimicrobial compounds [46,47]. σ^B^ is the general stress sigma factor of *B. subtilis* and is also involved in the transcription of general stress proteins induced by heat, salt, and oxygen stress [48]. σ^W^-dependent genes were distributed in clusters 4 and 9, and σ^B^-dependent genes were distributed in clusters 6, 7, and 9. Except for cluster 4, these clusters belong to the third group of clusters, which contains clusters 5, 6, 7, and 9 (genes that are turned on at the beginning of sporulation and then turned off in the middle). The genes for these stress-related sigma factor, *sigB* and *sigW*, were also up-regulated until 12 h and then down-regulated from 15 h (Table 4). The time point 12 h, where sporulation starts, is also a transition point in this study (Figure 1), and it was found that, if the growth of *B. subtilis* enters the transition point, stress responses may occur, even if the stresses are not induced from outside factors [7,49]. This study supports this view, showing that the σ^W^- or σ^B^-dependent genes that respond to stress are primarily turned on at the beginning of sporulation. Interestingly, cluster 4, whose expression was decreased from the transition point, also contains σ^W^-dependent genes: iron transporter genes from the *efeUOB* operon [50], general stress genes from the *yceCDEFGH* operon [51], and phage shock protein gene and unknown function genes from the *pspA*-*ydjGHI* operon [52]. It can be assumed that these genes have different expression patterns among the stress response genes because of the growth conditions used in the study. The results show that a spontaneous stress response may occur at the transition point, but even a general stress gene may not be induced at this time. 

For the spore-forming bacteria *B. subtilis*, nutrition limitation or high population density can trigger the complex process of sporulation [53]. During the growth of *B. subtilis* in this study, sporulation started from 12 h and progressed rapidly up to 18 h and then proceeded to 90% in 24 h (Figure 1). As expected, the GO term “sporulation (GO:0043934)” was enriched in clusters that belong to the third group, clusters 5, 6, and 7. Furthermore, in the GO analysis of these clusters, sporulation was significantly enriched with a relatively low *p*-value. Consistent with this, σ^E^, σ^F^, σ^G^, and σ^K^, which are related to gene expression during sporulation, are also included in clusters 6 and 7. The sporulation of this strain could be described as eight stages (stages 0–VII) based on morphological features [54,55]. In this study, we compared these sporulation stages with transcriptomic expression changes and analyzed them with the growth stage (Figure 5). Stages 0 and I are vegetative growth and axial filamentation, respectively. However, they are no longer distinguishable because of any mutant arrests at this stage [55], so we considered them together. In these stages, the cells start the sporulation with cannibalistic behavior (see the discussion below). The master regulator Spo0A is essential in transitioning from vegetative growth to the sporulation stage. The activation via the phosphorylation process from KinA/KinB to Spo0F, Spo0B, and Spo0A of the *spo0A* gene transcribed by the σ^H^-dependent promoter, the first activated sigma factor, signals the start of sporulation. In addition, phosphatase genes, such as the Rap family (*rapA*, *B*, *E*, and *H*) [56] and *Spo0E* [57], the cell division protein gene *racA* that helps chromosomes properly segregate [58], and the genes like *sirA* [59] and *sda* [60] that encode proteins for regulating the appropriate chromosome number, are expressed at these stages. All their expression levels were high at 8 h and/or 10 h, indicating that stages 0–I correspond to those before spores are formed. The activated Spo0A, Spo0A~P, regulates the *spoIIA* operon, which starts stage II. Genes, such as phosphatase gene *spoIIE* that dephosphorylates *SpoIIAA* [61], cell-division protein *ftsZ* that is required for septum formation [62], mother cell-specific sigma factor gene *sigE*, and protease gene *spoIIGA* that activates Pro-SigE [63], are expressed in stage II, including genes of *spoIIA* operon (anti-anti-sigma factor gene *spoIIAA*, anti-sigma factor gene *spoIIAB*, and forespore-specific sigma factor gene *sigF*). All of these genes expressed highly at 12 h, showing that stage II, at which asymmetric division occurs, matched the end of exponential growth. At stage III, the cell-wall hydrolase genes *spoIID* and *spoIIP* that are required for engulfment [64,65] are expressed. The membrane fission protein gene *fisB*, the *spoIIQ* gene, and the *spoIIIA* operon gene (*spoIIIAA*–*spoIIIAH*) that are required for the activation of σ^G^ [66] are also transcripted at this stage, where engulfment is processed. Their up-regulated expressed level from 10 h to 12 h indicates that stage III spans the end of exponential growth and the beginning of sporulation. Cortex and spore coat are produced and assembled at stages VI and V. Genes, such as spore coat morphogenetic protein gene *spoIVA*, DNA recombinase gene *spoIVCA* that is required for σ^K^ gene, and mother cell-specific sigma factor gene *sigKc*/*sigKn*, are related to cortex and spore coat. Most of these genes were highly expressed after 15 h (the formal part of sporulation), but some were up-regulated from 10 h (end of exponential growth). This result shows that the genes involved in stages VI and V could be expressed earlier. So, we decided to discuss spore coat genes in depth. 

σ^E^ and σ^F^ are the sigma factors related to early sporulation genes in the mother cell and forespore, respectively. At the same time, σ^K^ and σ^G^ are the sigma factors related to late sporulation genes in the mother cell and forespore, respectively. Additionally, many σ^K^-dependent genes were also included in cluster 8 of the fourth cluster group (genes that are up-regulated in the middle of sporulation). Notably, the expression of spore coat genes was clearly distinguished according to which sigma-factor-dependent RNA polymerase transcribes (Figure 6). The *B. subtilis* spore coat consists of two layers, an inner coat and outer coat. According to the sporulation process, the spore coat proteins are sequentially expressed in the mother cell [67]; however, it is not completely clear how many proteins make up the spore coat and how they are assembled. However, the results show that, no matter where the proteins exist, their transcription is affected only by σ^E^ or σ^K^, and there is no difference in the expression time point between them.

Cluster 8, where the GO term “acetoin metabolic process (GO:0045149)” was enriched (Supplementary File S2: Table S10), included some of the genes that are controlled by σ^L^, the acetoin dehydrogenase operon genes *acoABCL*. σ^L^ is known to be involved in degradative enzyme gene expression [68]. The acetoin is one of the extracellular products of *B*. *subtilis* [69]. Since it is reused as a carbon source after the cells enter to stationary phase and sporulation [70,71], the operon genes are up-regulated after 15 h. On the other hand, other genes belonging to σ^L^ regulon were not classified into any cluster, and even *sigL* (gene for σ^L^) was down-regulated from 12 h (Table 4). Similar to σ^L^, the regulons of other alternative sigma factors in *B*. *subtilis*, such as σ^I^, σ^M^, σ^X^, σ^Y^, and YlaA, did not show a consistent expression pattern (Supplementary File S1: Figure S11). Moreover, not only were the TPM values of *sigI*, *sigM*, *sigX*, *sigY*, and *ylaA* (gene for σ^I^, σ^M^, σ^X^, σ^Y^, and YlaA, respectively) much lower than those of other sigma factors (Table 4), there were also fewer genes belonging to their regulons. These results suggest that these sigma factors are minor compared to the other sigma factors described above (σ^A^, σ^B^, σ^D^, σ^E^, σ^F^, σ^G^, σ^K^, and σ^W^), and the role of other transcription regulators is essential for the regulation of the genes controlled by them.

Of particular interest, GO terms related to the lipid catabolic process were enriched only in cluster 10, which has a unique expression pattern. From these results, the genes related to the lipid catabolism of *B. subtilis* are expressed specifically throughout the entire sporulation when grown in a rich medium. The programmed cell death (PCD) system could be the reason for this specific expression. In *B. subtilis*, as in other eukaryotes or prokaryotes, a PCD system exists to delay sporulation, an energy-consuming and irreversible process [72]. The step-by-step expression of the gene involved in this system was well identified in our data. As shown in Figure 4c,d, the expression of *skfA* and *sdpC*, which play an essential role in the PCD system, peaked at 12 h and 10 h, respectively. In the Spo0A-active cell, genes encoded in the *skf* operon are expressed, and killing factors are produced and exported [73,74]. In previous transcriptome analysis conducted in LB with glucose [6], where sporulation was suppressed, there was no *skfA* expression changes during *B*. *subtilis* growth, indicating the PCD system occurs with spore formation. On the other hand, in both the Spo0A-active and -inactive cells, the *sdp* operon is turned on. SdpC (product of *sdpC*), a constituent protein of the *sdp* operon, increases the expression of the *fadNAE* operon, which encodes lipid catabolism enzymes through SdpR, which delays sporulation [75]. The *sdpR* and *fadNAE* operon gene expressions peaked at 12 h and 15 h, respectively (Supplementary File S2: Table S13). Therefore, it can be assumed that regulating the lipid catabolic process through the cascade of this sequential expression prevents sporulation triggers caused by a lack of energy. 

## 5. Conclusions

This transcriptome study provided a novel insight into the dynamic transcriptional response of each gene in *B. subtilis* DB104 during growth. The highest expressed genes and fold changes were identified around the transition point. Furthermore, the gene expression patterns were identified and DEG functions of were established through K-mean cluster analysis, GO enrichment analysis, the roles of alternative sigma factors, and their simultaneous application. The spore formation process is the most regulated in *B. subtilis* DB104, and most transcriptomic and metabolic changes occur with the entry of sporulation, named “the transition point”. Because sporulation is an essential program in this organism, many processes during growth, such as metabolism changes, stress-induced responses, and cannibalism, overlap with sporulation. The gene expression involved in each sporulation stage was compared and matched with a growth curve. Additionally, this research showed that the timing of expression of the spore coat gene is determined only by the sigma factor, regardless of its location. Our results also suggest that, among the sigma factors of *B. subtilis*, σ^A^, σ^B^, σ^D^, σ^E^, σ^F^, σ^G^, σ^K^, and σ^W^ play significant roles. Knowledge of transcriptional changes during the growth of *B*. *subtilis* DB104 will help to elucidate the expression and regulatory mechanisms of genes under various growth conditions and for the industrial application of this strain.

## Figures and Tables

**Table 1:** Transcripts per million (TPM) of the highest differentially expressed genes (DEGs).

Gene	Description	8 h	10 h	12 h	15 h	18 h	24 h	Total
*cotX*	spore coat protein (insoluble fraction)	290	62	72	21,516	75,558	18,170	115,668
*sspE*	small acid-soluble spore protein (gamma-type SASP)	140	382	17,439	68,242	10,155	17,838	114,196
*yczN*	putative spore and germination protein	590	673	851	685	11,545	97,628	111,972
*gerE*	transcriptional regulator of late spore coat genes	382	149	123	17,071	50,095	32,767	100,587
*cotY*	outer spore coat protein (crust layer, insoluble fraction)	8	78	236	21,147	65,667	10,293	97,429
*cotZ*	spore coat protein (insoluble fraction, crust layer)	9	135	364	19,373	55,345	8361	83,588
*sdpC*	precursor of killing factor SdpC	15,175	32,303	13,413	2336	980	6666	70,873
*skfA*	sporulation killing factor A	3653	16,990	37,157	6531	2708	3054	70,093
*cotV*	spore coat protein (insoluble fraction)	254	44	35	9404	38,992	17,226	65,956
*cgeB*	maturation of the outermost layer of the spore	349	122	78	4675	22,332	35,830	63,386
*cgeA*	spore outermost layer component	319	151	108	3299	17,995	41,382	63,254
*cotD*	spore coat protein (inner)	112	39	54	11,744	38,217	10,030	60,197
*yczM*	putative type I toxin	314	482	240	486	7142	49,157	57,820
*sspB*	small acid-soluble spore protein (beta-type SASP)	3	84	8082	35,591	2720	4286	50,766
*safA*	morphogenetic protein associated with SpoVID	6	5516	18,998	17,402	3633	4705	50,260

**Table 2:** The top 10 most up- and down-regulated DEGs according to fold change.

Gene	Description	Pairwise Time	Fold Change	*p*-Value	Adj. *p*-Value ^a^
*cotD*	spore coat protein (inner)	15 h/12 h	308.74	2.28 × 10^−43^	4.45 × 10^−40^
*spoIIID*	transcriptional regulator (stage III sporulation)	10 h/8 h	257.78	2.07 × 10^−38^	8.08 × 10^−35^
*yqfX*	conserved protein of unknown function expressed in germinating spores	12 h/10 h	236.64	1.16 × 10^−25^	1.13 × 10^−22^
*spmH*	glucose-1-phosphate cytidylyltransferase (sporulation)	15 h/12 h	223.84	1.08 × 10^−32^	3.84 × 10^−30^
*yhdB*	conserved hypothetical protein	12 h/10 h	217.38	1.10 × 10^−18^	3.92 × 10^−16^
*ypzD*	putative germination protein	15 h/12 h	210.05	1.35 × 10^−31^	4.41 × 10^−29^
*cotNE*	inner spore coat protein	12 h/10 h	203.64	4.51 × 10^−11^	2.75 × 10^−9^
*yurS*	conserved protein of unknown function	15 h/12 h	175.04	2.15 × 10^−24^	4.41 × 10^−22^
*cotG*	spore morphogenetic protein	15 h/12 h	172.24	5.10 × 10^−45^	1.99 × 10^−41^
*sppO*	spore protein cse15	10 h/8 h	166.91	9.57 × 10^−20^	1.97 × 10^−17^
*gsiB*	general stress protein glucose starvation induced	15 h/12 h	−16.30	1.02 × 10^−9^	3.60 × 10^−8^
*frlO*	fructose amino acid-binding lipoprotein	12 h/10 h	−16.78	2.48 × 10^−10^	1.31 × 10^−8^
*cheY*	regulator of chemotaxis and motility	10 h/8 h	−17.47	2.11 × 10^−10^	4.17 × 10^−9^
*gbsB*	choline dehydrogenase	12 h/10 h	−20.41	2.69 × 10^−5^	2.07 × 10^−4^
*frlN*	fructose-amino acid permease	12 h/10 h	−20.81	2.75 × 10^−11^	1.76 × 10^−9^
*frlM*	fructose-amino acid permease	12 h/10 h	−25.96	1.06 × 10^−12^	9.63 × 10^−11^
*gbsA*	glycine betaine aldehyde dehydrogenase, NAD+-dependent	12 h/10 h	−36.86	3.30 × 10^−7^	5.59 × 10^−6^
*gabT*	4-aminobutyrate aminotransferase	10 h/8 h	−39.08	3.16 × 10^−18^	3.75 × 10^−16^
*xpt*	xanthine phosphoribosyltransferase	10 h/8 h	−48.69	2.65 × 10^−18^	3.30 × 10^−16^
*pbuX*	xanthine permease	10 h/8 h	−88.52	1.81 × 10^−19^	3.07 × 10^−17^

^a^ *p*-value after FDR correction.

**Table 3:** The top 10 KEGG pathways of up- or down-regulated DEGs.

Pathway ID	KEGG Pathway	10 h/8 h	12 h/10 h	15 h/12 h	18 h/15 h	24 h/18 h
Up-regulated						
bsu01100	Metabolic pathways	111	120	87	39	0
bsu01110	Biosynthesis of secondary metabolites	49	53	41	21	0
bsu01120	Microbial metabolism in diverse environments	31	36	29	0	0
bsu00500	Starch and sucrose metabolism	11	15	11	7	0
bsu01250	Biosynthesis of nucleotide sugars	8	10	7	8	0
bsu01230	Biosynthesis of amino acids	8	10	7	8	0
bsu00541	O-Antigen nucleotide sugar biosynthesis	0	0	20	0	11
bsu02010	ABC transporters	0	0	0	11	10
bsu00520	Amino sugar and nucleotide sugar metabolism	0	12	9	0	0
bsu00650	Butanoate metabolism	8	10	0	0	0
Down-regulated						
bsu01100	Metabolic pathways	0	0	73	61	25
bsu01120	Microbial metabolism in diverse environments	38	34	0	23	10
bsu02040	Flagellar assembly	31	22	0	0	0
bsu02010	ABC transporters	26	26	0	0	0
bsu02030	Bacterial chemotaxis	31	11	0	0	0
bsu01230	Biosynthesis of amino acids	0	29	0	0	0
bsu02020	Two-component system	28	0	0	0	0
bsu00230	Purine metabolism	17	0	10	0	0
bsu00650	Butanoate metabolism	7	6	6	0	0
bsu00020	Citrate cycle (TCA cycle)	8	10	0	0	0

**Table 4:** Transcripts per million (TPM) of sigma-factor-encoding genes.

Gene	Description	8 h	10 h	12 h	15 h	18 h	24 h
*sigA*	RNA polymerase major sigma-43 factor (sigma-A)	984	863	1747	757	459	593
*sigB*	RNA polymerase sigma-37 factor (sigma-B)	383	618	317	105	120	120
*sigD*	RNA polymerase sigma-28 factor (sigma-D)	352	33	7	3	2	10
*sigE*	RNA polymerase sporulation-specific sigma-29 factor (sigma-E)	95	565	275	49	21	62
*sigF*	RNA polymerase sporulation-specific sigma factor (sigma-F)	2015	8531	6968	1215	741	873
*sigG*	RNA polymerase sporulation-specific sigma factor (sigma-G)	152	1073	1159	1444	140	287
*sigI*	RNA polymerase sigma factor (heat stress responsive)	8	10	10	6	4	17
*sigKc*	RNA polymerase sporulation-specific sigma-K factor precursor (C-terminal fragment)	1	5	112	426	71	66
*sigKn*	RNA polymerase sporulation-specific sigma-K factor precursor (N-terminal half)	9	37	231	538	87	102
*sigL*	RNA polymerase sigma-54 factor (sigma-L)	198	238	156	97	39	124
*sigM*	RNA polymerase ECF (extracytoplasmic function)-type sigma factor (sigma-M)	19	17	17	8	4	20
*sigX*	RNA polymerase ECF (extracytoplasmic function)-type sigma factor (sigma-X)	35	42	22	22	15	40
*sigW*	RNA polymerase ECF (extracytoplasmic function)-type sigma factor (sigma-W)	1304	1211	1100	254	150	437
*sigY*	RNA polymerase ECF (extracytoplasmic function)-type sigma factor (sigma Y)	8	14	16	9	8	18
*ylaA*	conserved protein of unknown function	0	0	1	0	0	1

## Data Availability

The datasets supporting the conclusions of this article are included within the article as well as their additional files. The raw sequence data have been submitted to the Sequence Read Archive (SRA, https://www.ncbi.nlm.nih.gov/sra, accessed on 13 February 2023) under Bioproject accession number PRJNA934277 and reference Biosample accession number SAMN33269663—80.

## Supplementary Materials

The following supporting information can be downloaded at: https://www.mdpi.com/article/10.3390/microorganisms11081928/s1. Supplementary File S1. Figure S1: Observation of sporulation by optical microscopy at each time point, Figure S2: Gene ontology (GO) term networks of differentially expressed genes involved in cluster 1, Figure S3: Gene ontology (GO) term networks of differentially expressed genes involved in cluster 2, Figure S4: Gene ontology (GO) term networks of differentially expressed genes involved in cluster 3, Figure S5: Gene ontology (GO) term networks of differentially expressed genes involved in cluster 4, Figure S6: Gene ontology (GO) term networks of differentially expressed genes involved in cluster 5, Figure S7: Gene ontology (GO) term networks of differentially expressed genes involved in cluster 6, Figure S8: Gene ontology (GO) term networks of differentially expressed genes involved in cluster 7, Figure S9: Gene ontology (GO) term networks of differentially expressed genes involved in cluster 8, Figure S10: Gene ontology (GO) term networks of differentially expressed genes involved in cluster 10, Figure S11: Expression patterns of the genes included in each regulon. Supplementary File S2; Table S1: Summary of RNA-seq and the reads mapped to the genome of Bacillus subtilis subsp. subtilis str. 168, Table S2: Primers used for RT-qPCR analysis. Table S3: Gene ontology (GO) enrichment analysis of cluster 1, Table S4: Gene ontology (GO) enrichment analysis of cluster 2, Table S5: Gene ontology (GO) enrichment analysis of cluster 3, Table S6: Gene ontology (GO) enrichment analysis of cluster 4, Table S7: Gene ontology (GO) enrichment analysis of cluster 5, Table S8: Gene ontology (GO) enrichment analysis of cluster 6, Table S9: Gene ontology (GO) enrichment analysis of cluster 7, Table S10: Gene ontology (GO) enrichment analysis of cluster 8, Table S11: Gene ontology (GO) enrichment analysis of cluster 10, Table S12: Transcripts per millions mapped reads (TPM) values of DEGs involved in each structure, Table S13: Transcripts per millions mapped reads (TPM) values of DEGs involved in each operon. Supplementary File S3; List of the differentially expressed genes in clusters 1 to 10.
