# Supplementary material for: Time-Course Transcriptome Analysis of Bacillus subtilis DB104 during Growth
Source: Microorganisms. 2023 Jul 28;11(8):1928. doi: 10.3390/microorganisms11081928 (PMC10458515; doi:10.3390/microorganisms11081928)
Supplement: Supplementary file 1 [file microorganisms-11-01928-s001.zip › supplementary File S1.pdf]

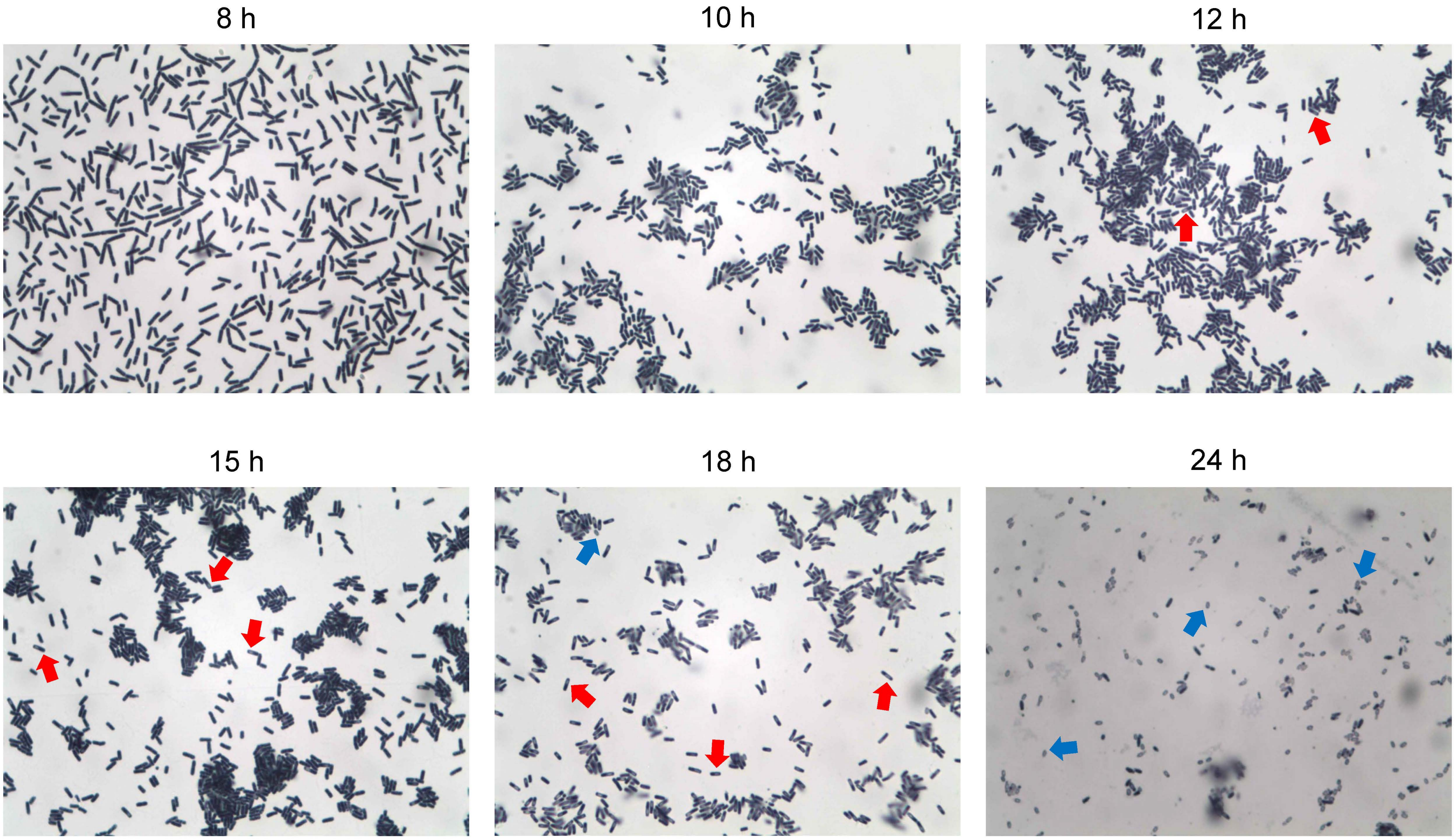

Figure S1 Observation of sporulation by optical microscopy at each time point. Red arrows indicate endospores and blue arrows indicate free spores.

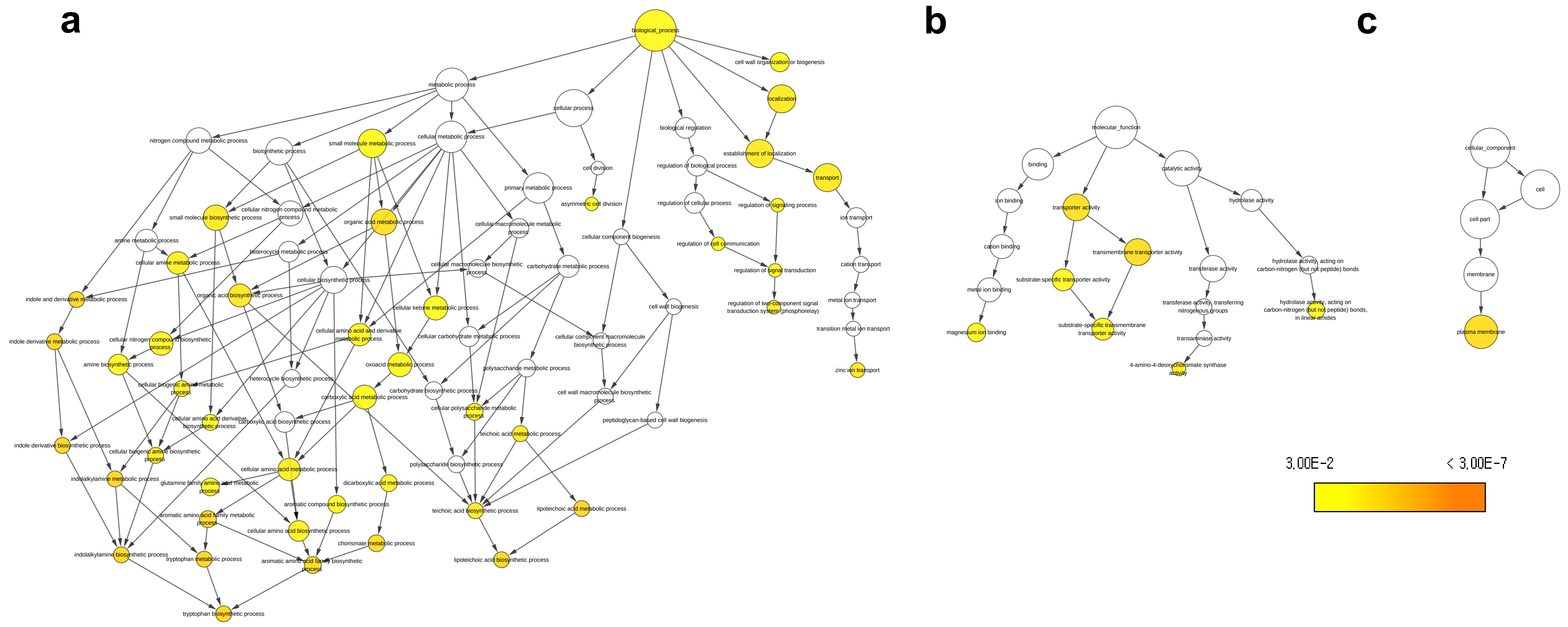

Figure S2 Gene ontology (GO) term networks of differentially expressed genes involved in cluster 1. Networks were generated by Cytoscape BiNGO. All significantly enriched GO terms in a biological process, b molecular function, and c cellular component are presented. Node size represents proportional to the number of targets in GO. Nodes represented in white were not significantly enriched, while a deeper shade of yellow correlated with higher significance. Threshold of hypergeometric distribution of the functional annotation was set at a corrected p-value < 0.03.

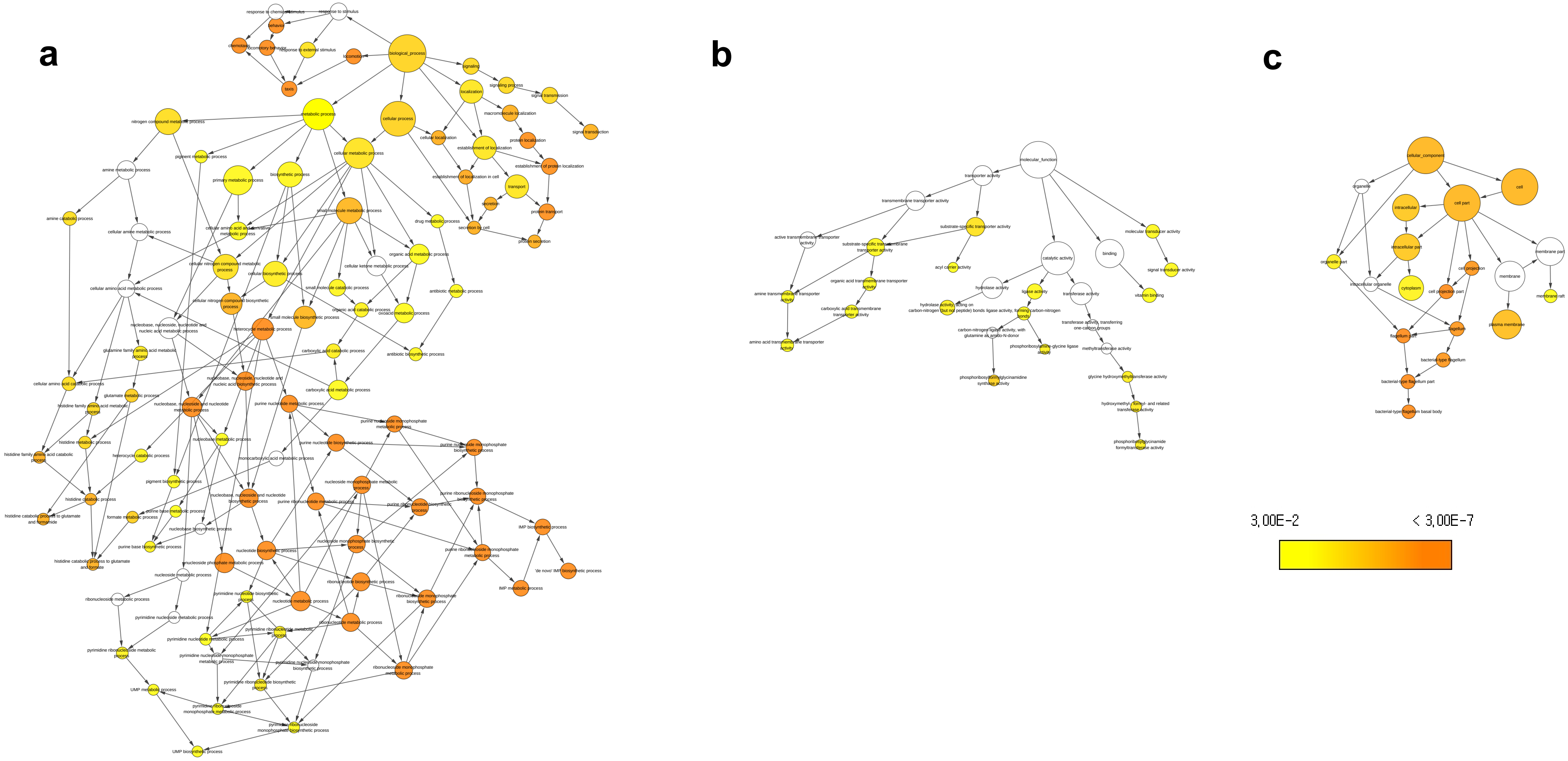

Figure S3 Gene ontology (GO) term networks of differentially expressed genes involved in cluster 2. Networks were generated by Cytoscape BiNGO. All significantly enriched GO terms in a biological process, b molecular function, and c cellular component are presented. Node size represents proportional to the number of targets in GO. Nodes represented in white were not significantly enriched, while a deeper shade of yellow correlated with higher significance. Threshold of hypergeometric distribution of the functional annotation was set at a corrected p-value < 0.03.

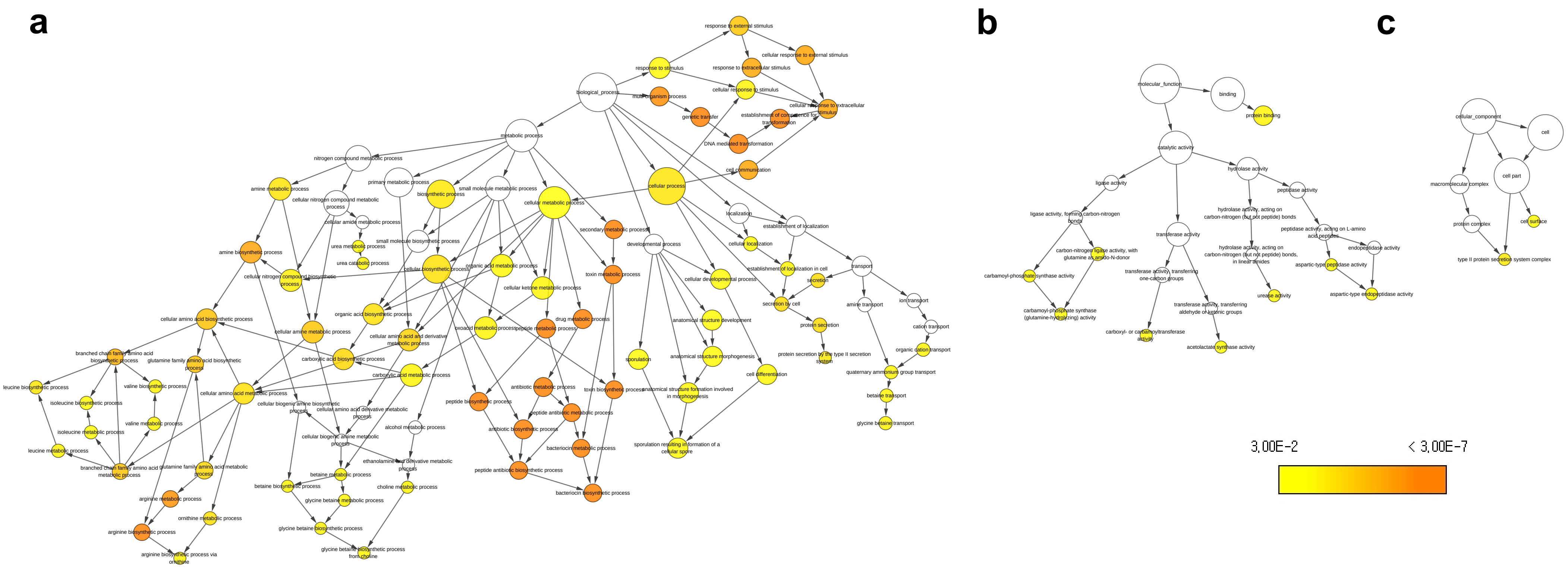

Figure S4 Gene ontology (GO) term networks of differentially expressed genes involved in cluster 3. Networks were generated by Cytoscape BiNGO. All significantly enriched GO terms in a biological process, b molecular function, and c cellular component are presented. Node size represents proportional to the number of targets in GO. Nodes represented in white were not significantly enriched, while a deeper shade of yellow correlated with higher significance. Threshold of hypergeometric distribution of the functional annotation was set at a corrected p-value < 0.03.

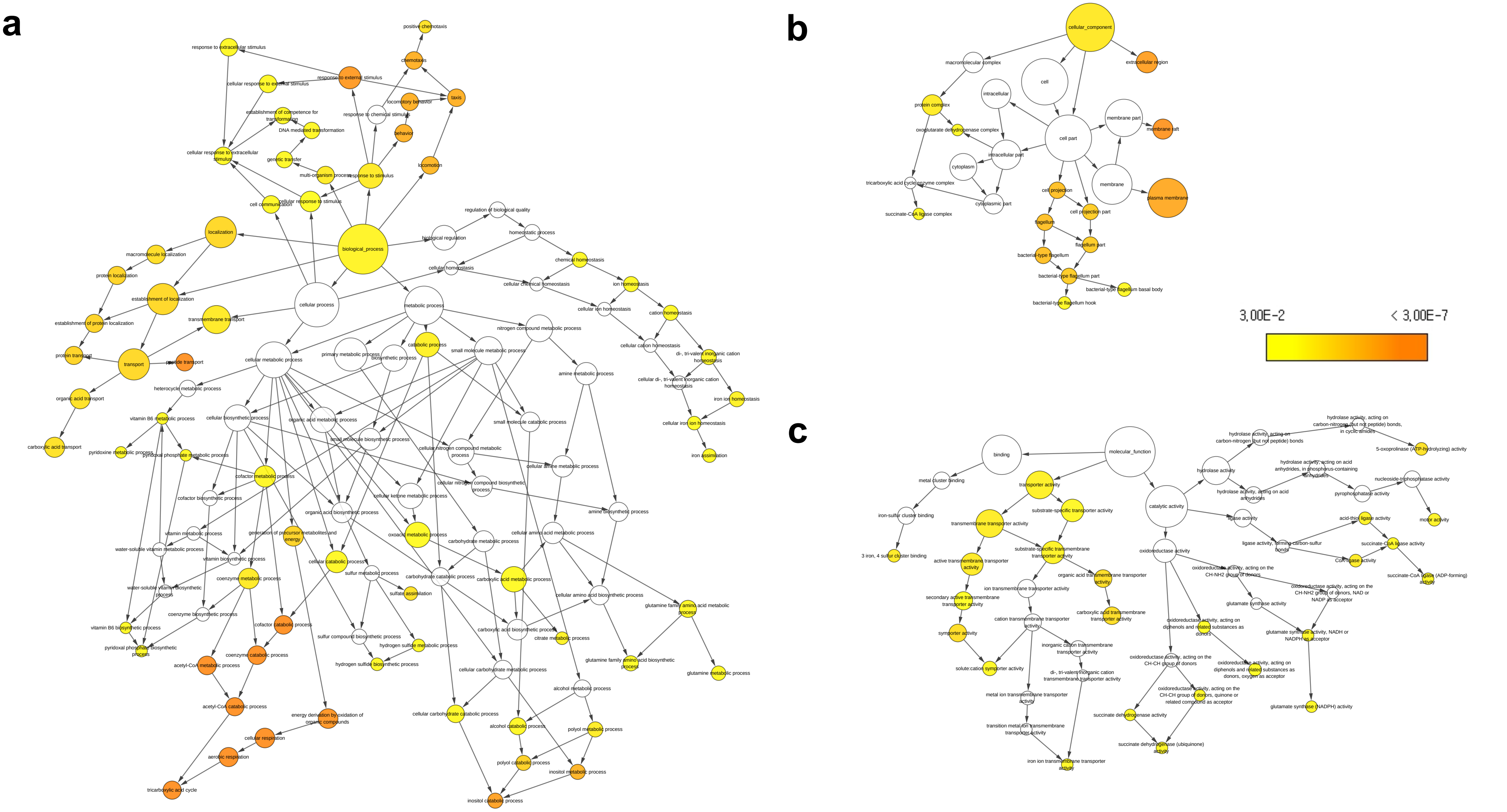

Figure S5 Gene ontology (GO) term networks of differentially expressed genes involved in cluster 4. Networks were generated by Cytoscape BiNGO. All significantly enriched GO terms in a biological process, b cellular component, and c molecular function are presented. Node size represents proportional to the number of targets in GO. Nodes represented in white were not significantly enriched, while a deeper shade of yellow correlated with higher significance. Threshold of hypergeometric distribution of the functional annotation was set at a corrected p-value < 0.03.

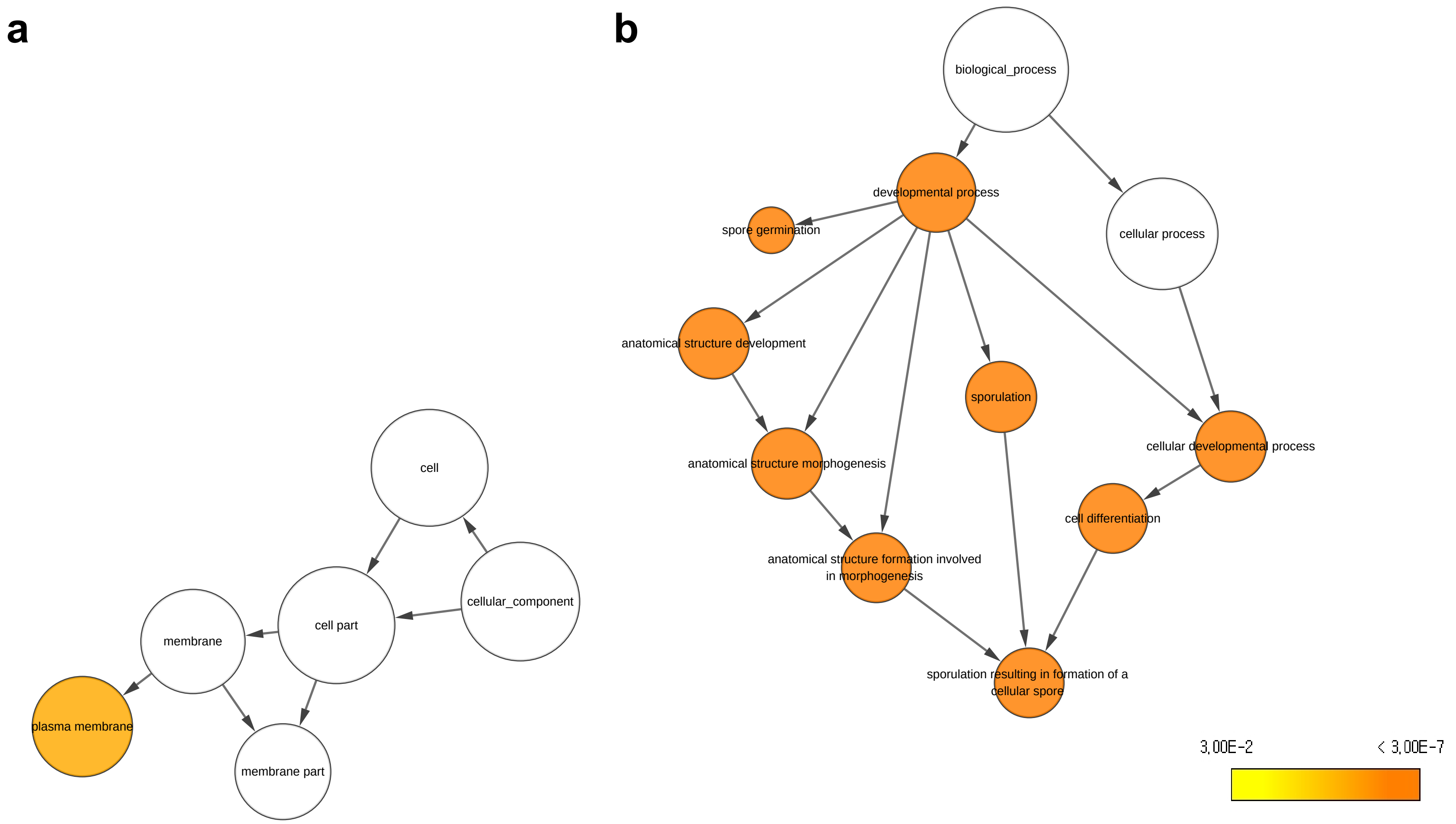

Figure S6 Gene ontology (GO) term networks of differentially expressed genes involved in cluster 5. Networks were generated by Cytoscape BiNGO. All significantly enriched GO terms in a cellular component and b biological process are presented. Node size represents proportional to the number of targets in GO. Nodes represented in white were not significantly enriched, while a deeper shade of yellow correlated with higher significance. Threshold of hypergeometric distribution of the functional annotation was set at a corrected p-value < 0.03.

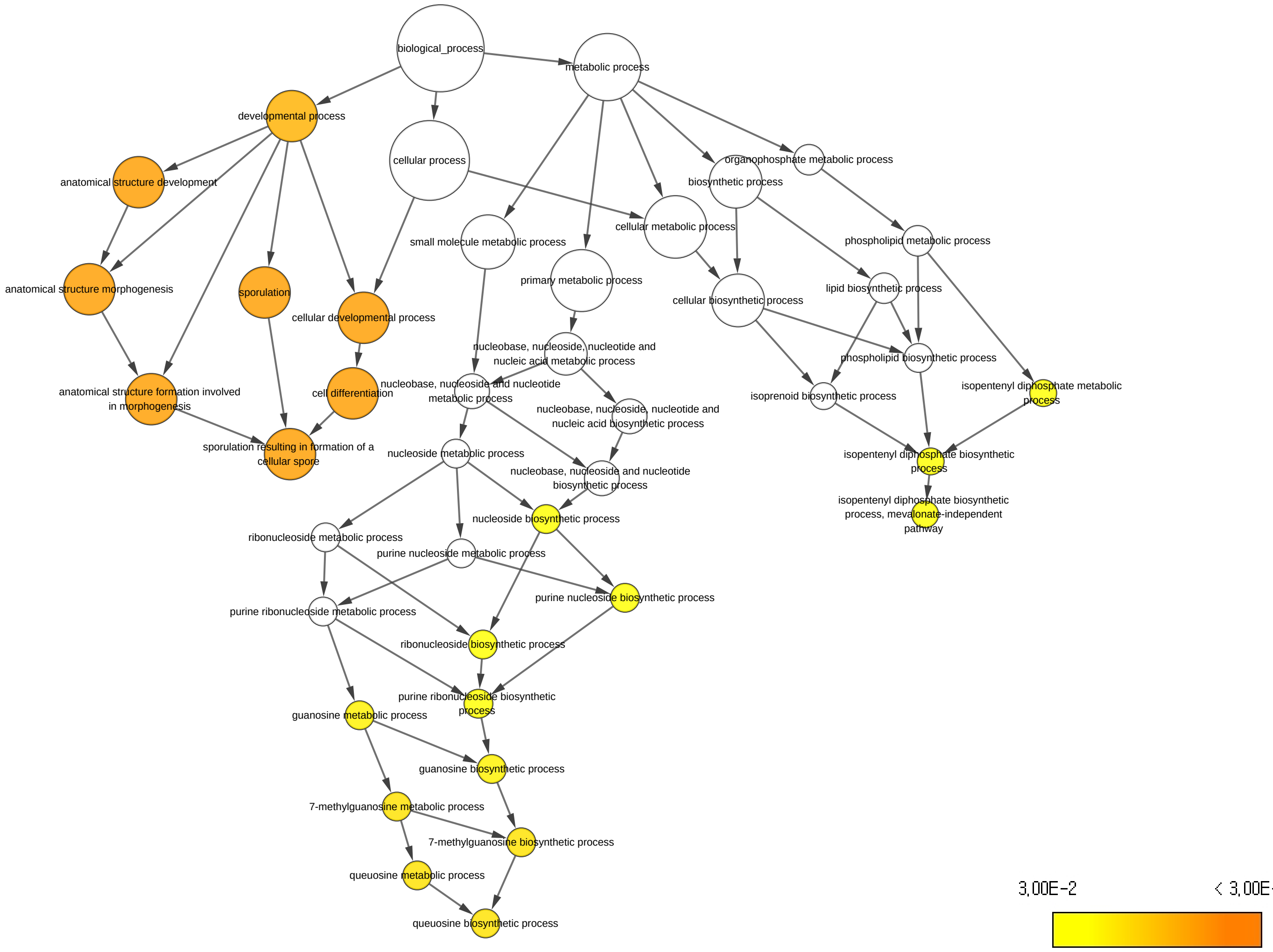

Figure S7 Gene ontology (GO) term networks of differentially expressed genes involved in cluster 6. Networks were generated by Cytoscape BiNGO. All significantly enriched GO terms in biological process is presented. Node size represents proportional to the number of targets in GO. Nodes represented in white were not significantly enriched, while a deeper shade of yellow correlated with higher significance. Threshold of hypergeometric distribution of the functional annotation was set at a corrected p-value < 0.03.

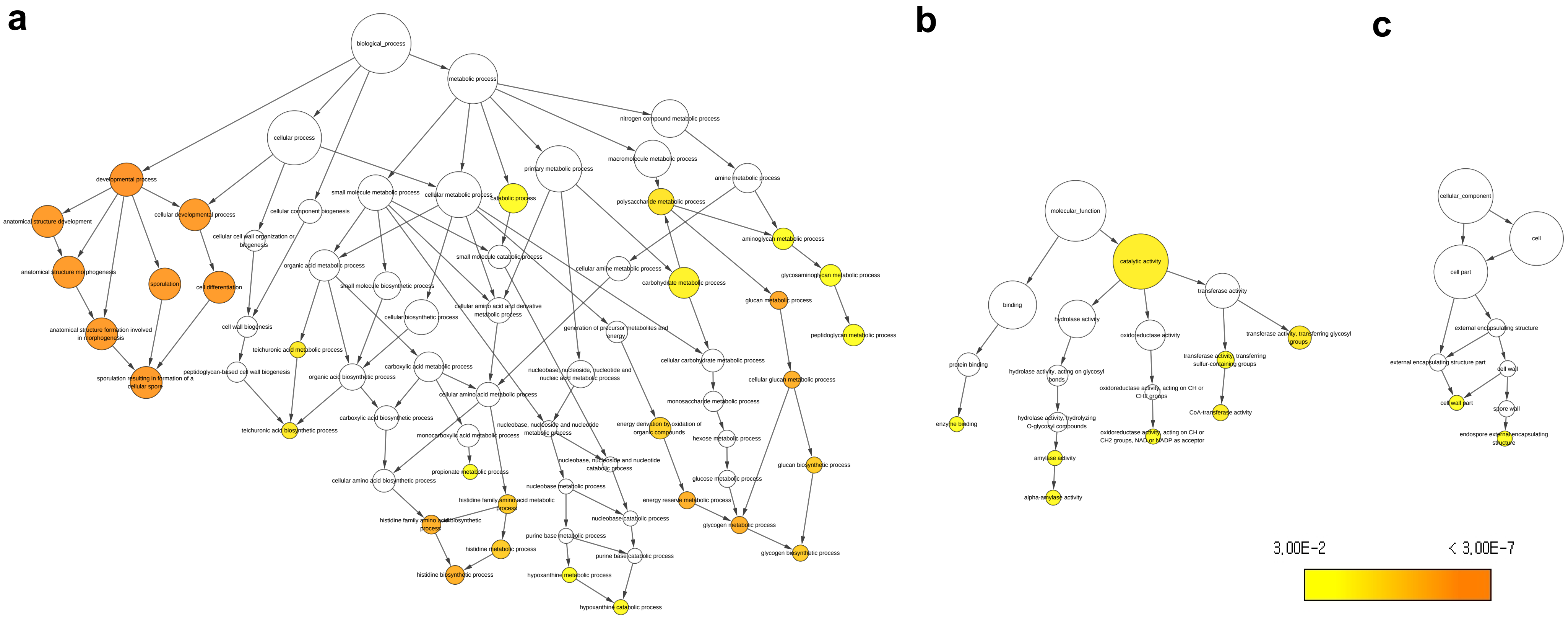

Figure S8 Gene ontology (GO) term networks of differentially expressed genes involved in cluster 7. Networks were generated by Cytoscape BiNGO. All significantly enriched GO terms in a biological process, b cellular component, and c molecular function are presented. Node size represents proportional to the number of targets in GO. Nodes represented in white were not significantly enriched, while a deeper shade of yellow correlated with higher significance. Threshold of hypergeometric distribution of the functional annotation was set at a corrected p-value < 0.03.

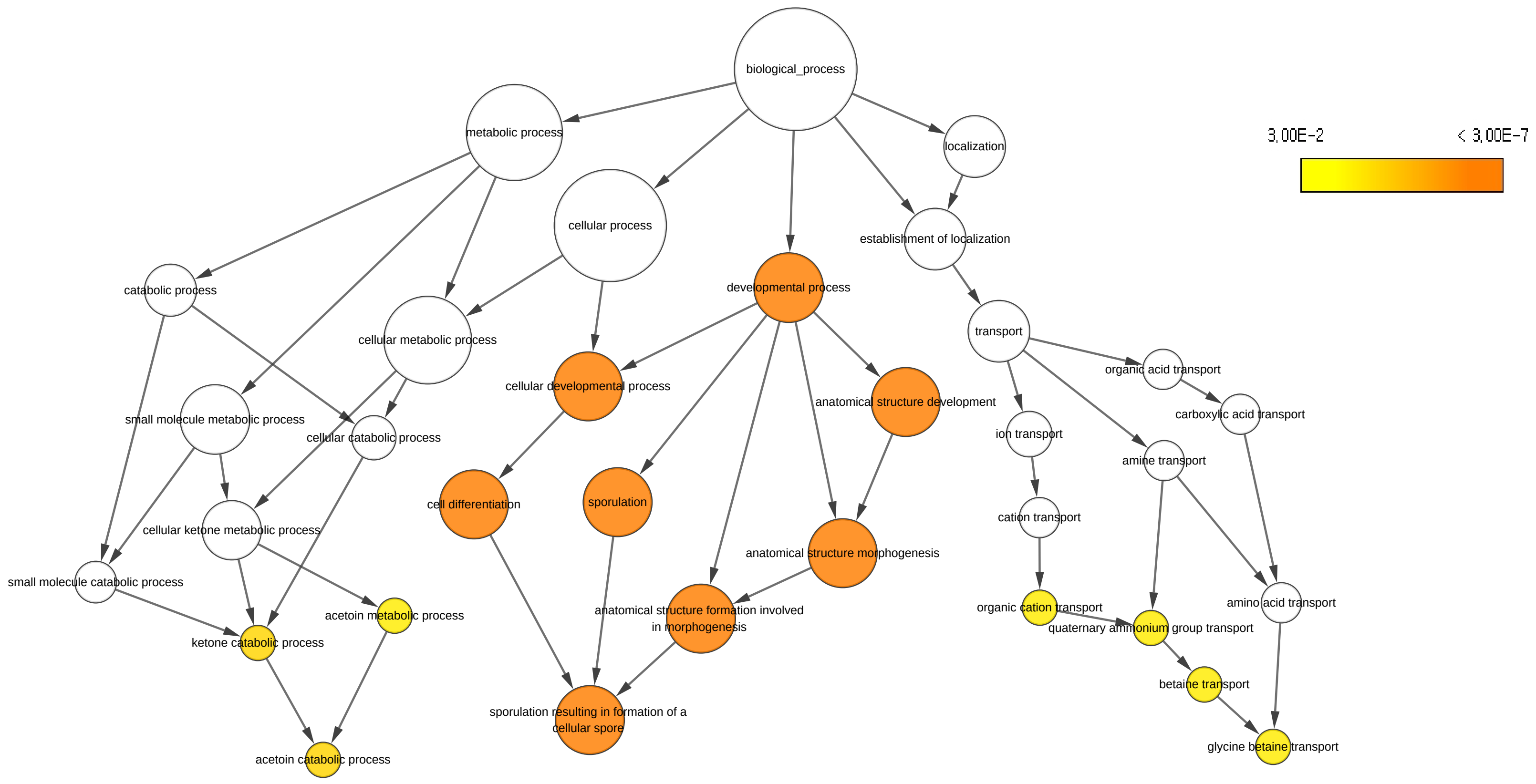

Figure S9 Gene ontology (GO) term networks of differentially expressed genes involved in cluster 8. Networks were generated by Cytoscape BiNGO. All significantly enriched GO terms in biological process are presented. Node size represents proportional to the number of targets in GO. Nodes represented in white were not significantly enriched, while a deeper shade of yellow correlated with higher significance. Threshold of hypergeometric distribution of the functional annotation was set at a corrected p-value < 0.03.

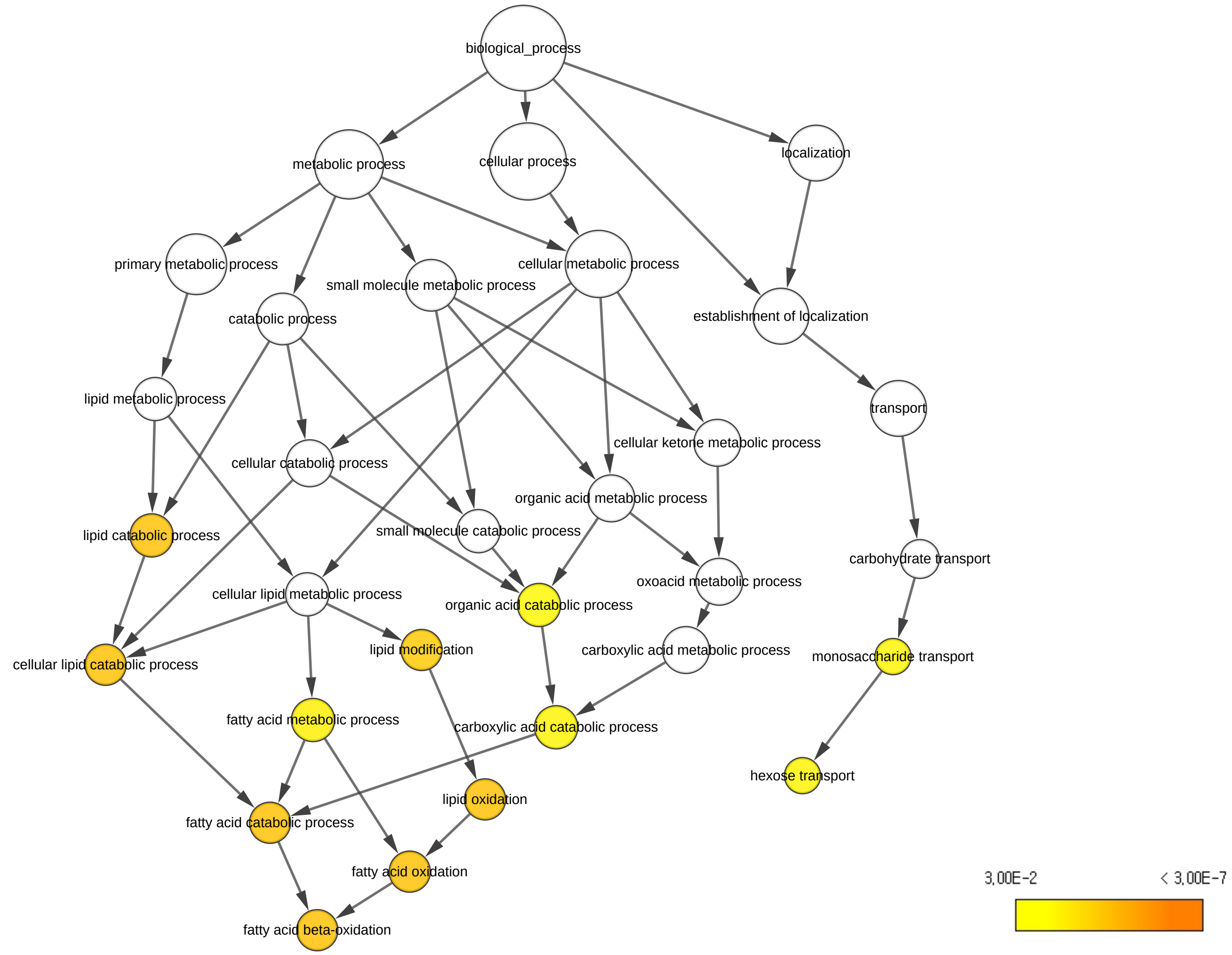

Figure S10 Gene ontology (GO) term networks of differentially expressed genes involved in cluster 10. Networks were generated by Cytoscape BiNGO. All significantly enriched GO terms in biological process is presented. Node size represents proportional to the number of targets in GO. Nodes represented in white were not significantly enriched, while a deeper shade of yellow correlated with higher significance. Threshold of hypergeometric distribution of the functional annotation was set at a corrected p-value < 0.03.

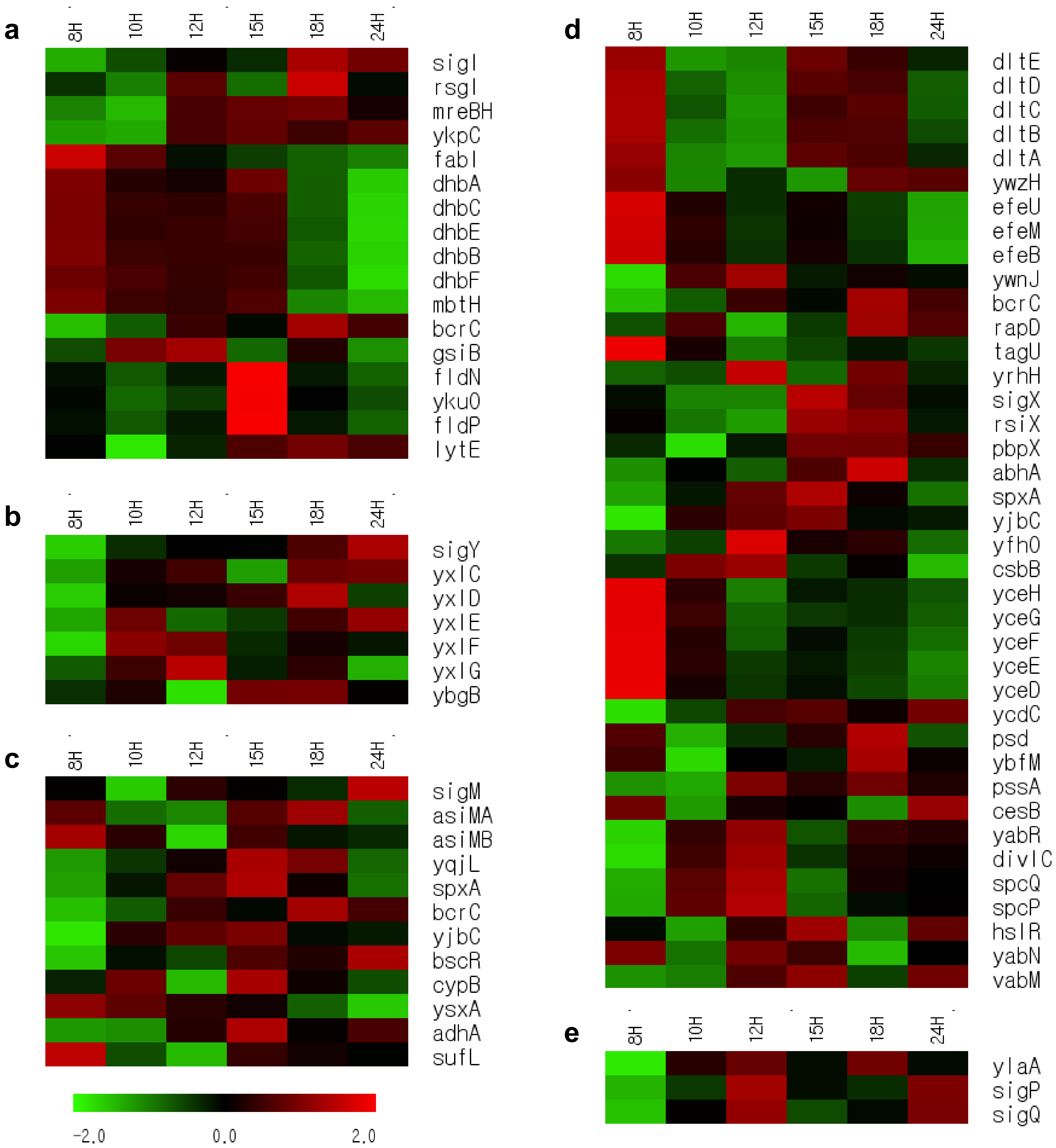

Figure S11 Expression patterns of gene included in **a** SigI regulon, **b** SigY regulon, **c** SigM regulon, **d** SigX regulon, and **e** YlaA regulon. Gene expression is visualized as the Z-score. The red and green color represents the up-regulated and down-regulated gene, respectively.
